# Isradipine therapy in *Cacna1d^Ile772Met/+^* mice ameliorates primary aldosteronism and neurologic abnormalities

**DOI:** 10.1172/jci.insight.162468

**Published:** 2023-10-23

**Authors:** Gabriel Stölting, Hoang An Dinh, Marina Volkert, Nicole Hellmig, Julia Schewe, Luise Hennicke, Eric Seidel, Herbert Oberacher, Junhui Zhang, Richard P. Lifton, Iris Urban, Melissa Long, Marion Rivalan, Timothy Nottoli, Ute I. Scholl

**Affiliations:** 1 Center of Functional Genomics, Berlin Institute of Health at Charité – Universitätsmedizin Berlin, Berlin, Germany.; 2 Institute of Legal Medicine and Core Facility Metabolomics, Medical University of Innsbruck, Innsbruck, Austria.; 3 Department of Genetics and Howard Hughes Medical Institute, Yale University School of Medicine, New Haven, Connecticut, USA.; 4 Laboratory of Human Genetics and Genomics, The Rockefeller University, New York, New York, USA.; 5 Transgenic Technologies and; 6 Animal Behavior Phenotyping Facility (ABPF), Charité – Universitätsmedizin Berlin, Berlin, Germany.; 7 Section of Comparative Medicine, Yale Genome Editing Center, Yale University School of Medicine, New Haven, Connecticut, USA.

**Keywords:** Endocrinology, Neuroscience, Hypertension, Mouse models, Neurological disorders

## Abstract

Somatic gain-of-function mutations in the L-type calcium channel Ca_V_1.3 (*CACNA1D* gene) cause adrenal aldosterone-producing adenomas and micronodules. De novo germline mutations are found in a syndrome of primary aldosteronism, seizures, and neurologic abnormalities (PASNA) as well as in autism spectrum disorder. Using CRISPR/Cas9, we here generated mice with a *Cacna1d* gain-of-function mutation found in both adenomas and PASNA syndrome (*Cacna1d^Ile772Met/+^*). These mice show reduced body weight and increased mortality from weaning to approximately 100 days of age. Male mice do not breed, likely due to neuromotor impairment, and the offspring of female mice die perinatally, likely due to lack of maternal care. Mice generated by in vitro fertilization showed elevated intracellular calcium in the aldosterone-producing zona glomerulosa, an elevated aldosterone/renin ratio, and persistently elevated serum aldosterone on a high-salt diet as signs of primary aldosteronism. Anesthesia with ketamine and xylazine induced tonic-clonic seizures. Neurologic abnormalities included hyperlocomotion, impaired performance in the rotarod test, impaired nest building, and slight changes in social behavior. Intracellular calcium in the zona glomerulosa, aldosterone levels, and rotarod performance responded to treatment with the calcium channel blocker isradipine, with implications for the therapy of patients with aldosterone-producing lesions and with PASNA syndrome.

## Graphical abstract

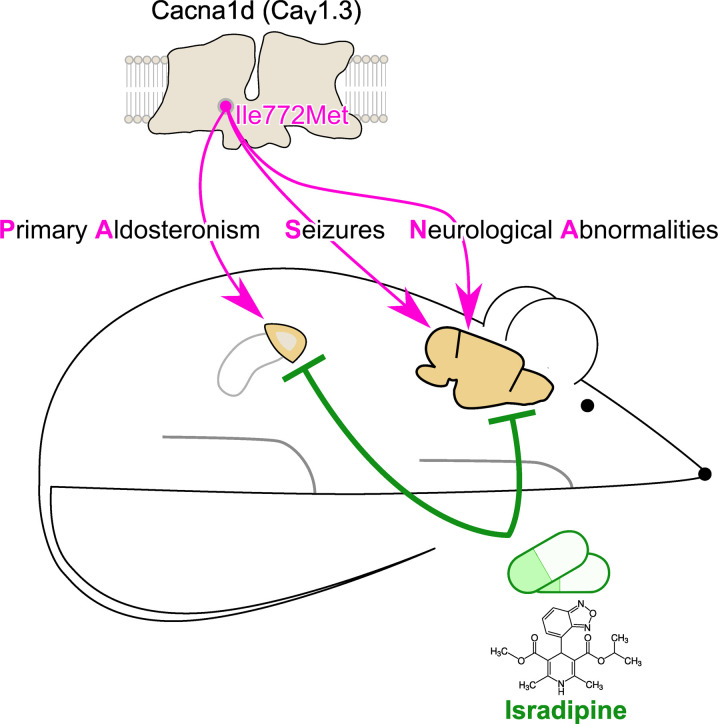

## Introduction

Hypertension affects approximately one-third of the adult population (1) and is a major contributor to morbidity and mortality worldwide (2). Most cases are considered primary. A causative underlying disease (secondary hypertension) is found in approximately 10% of patients. Primary aldosteronism (PA), the excessive production of the adrenal steroid hormone aldosterone, is the most common cause of secondary hypertension. PA affects at least 50 million people worldwide (3), but recent studies have found evidence of partially autonomous aldosterone production in > 10% of normotensive individuals and > 20% of patients with hypertension (4). PA can be caused by either aldosterone-producing adenomas (benign tumors), multiple aldosterone-producing micronodules (smaller lesions), or diffuse hyperplasia of the adrenal gland (5). Aldosterone-producing micronodules also occur in healthy individuals (6). More than 95% of aldosterone-producing adenomas and 60%–80% of micronodules from patients with PA without adenoma carry somatic mutations in known disease genes (7). In 2013, we and others discovered heterozygous somatic gain-of-function mutations in the *CACNA1D* gene, encoding voltage-gated L-type calcium channel Ca_V_1.3, as a cause of aldosterone-producing adenomas (8, 9). These mutations account for ~20% of tumors in individuals of recent European ancestry, ~40% in those of recent African ancestry, and ~15% in those of Asian ancestry. For unknown reasons, they are more prevalent in men than in women (10). *CACNA1D* is the most frequently mutated gene in adenomas from those of recent African ancestry and the second most frequently mutated gene (after *KCNJ5*; ref. 11) in those of recent European ancestry and Asians (10). In addition, ~60% of micronodules from patients with PA without adenomas (12, 13) and ~25% of micronodules from apparently healthy adrenal glands (6, 14) carry somatic *CACNA1D* mutations.

*CACNA1D* is expressed in a variety of tissues, including adrenal gland, heart, brain, inner ear, and pancreatic islets (15, 16). Beyond the relevance of somatic mutations in sporadic PA, germline loss-of-function mutations cause bradycardia and congenital deafness (15), while gain-of-function *CACNA1D* mutations cause a rare syndrome of PA, seizures, and neurologic abnormalities (PASNA). We previously identified 2 individuals with PASNA due to de novo mutations at positions also mutated in aldosterone-producing adenomas (p.Gly403Asp and p.Ile770Met). Besides PA and hypertension, both subjects had a seizure disorder and several neurologic symptoms (including apparent cerebral palsy). Variably associated symptoms included cortical blindness, spastic quadriplegia, a ventricular septum defect, second-degree heart block, and transient hypoglycemia (8). Several additional patients with gain-of-function *CACNA1D* mutations have been reported (17). In some, symptoms are apparently limited to autism spectrum disorder without evidence of seizures or endocrine abnormalities (18–20). In another case, hyperinsulinemic hypoglycemia and hypotonia were predominant, and no evidence of PA was found (21). Thus, there appears to be a spectrum of different disease severities associated with de novo *CACNA1D* mutations. This striking variability may be due to differential effects of individual variants on calcium entry (17), differences in expression levels, and calcium signaling in the numerous organs involved in disease pathology and/or differences in genetic background.

PA-associated mutations in Ca_V_1.3 cause activation of the channel at more hyperpolarized (more negative) potentials and, in some cases, impaired inactivation of the channel (8), leading to increased calcium influx and activation of calcium signaling. This leads to elevated aldosterone production in the adrenal gland and likely also accounts for neurologic abnormalities and increased pancreatic insulin secretion.

Unlike in oncology, where personalized therapies based on molecular profiling of tumors have changed outcomes of patients with several malignancies (22), hypertension treatment is still largely based on few clinical characteristics and trial-and-error in the individual case (23–26). Most patients with PA remain undiagnosed and are not treated appropriately (27), and genetic characteristics of underlying lesions are not accounted for in therapeutic decisions.

To better understand the pathophysiology of *CACNA1D* mutations in sporadic PA and PASNA syndrome and to explore the pharmacologic reversibility of both adrenal and neurologic phenotypes, we generated of a gain-of-function *Cacna1d* animal model. Our model carries a mutation equivalent to the human Ile770Met mutation found in aldosterone-producing adenomas and in PASNA syndrome (*Cacna1d^Ile772Met/+^*). We chose the calcium channel blocker isradipine for this study, as it combines high affinity for Ca_V_1.3 with high brain penetration (28, 29).

## Results

### Generation of Cacna1d^Ile772Met/+^ mice.

**Figure 1:**
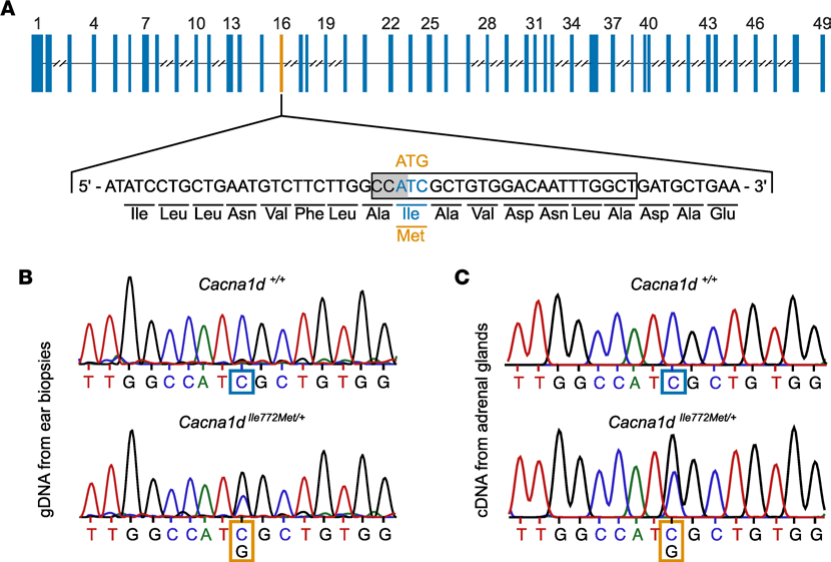
Generation of *Cacna1d^Ile772Met/+^* mice. (**A**) Topology of the *Cacna1d* gene (isoform ENSMUST00000238504.1) with Exons as blue boxes and intronic segments as thin lines. Introns longer than 2,000 bp are truncated. The sequence encoding the first 18 amino acids of exon 16 (encoding Ile772) is shown below. Substitution of ATG (Met; orange) for ATC (Ile; blue) was performed using CRISPR/Cas9 editing. The PAM site (gray background) as well as the gRNA (black box) used for mutagenesis are highlighted. (**B**) Sequencing of genomic DNA obtained from ear biopsies shows the heterozygous mutation. (**C**) Sequencing of cDNA from adrenal gland demonstrates expression of the mutant RNA.

The amino acids surrounding human Ca_V_1.3 Ile770 affected by a mutation in PA are completely conserved in mice (8). The homologous mouse residue, Ile772 (NM_028981, NP_083257), is encoded on exon 16 of the mouse *Cacna1d* gene (Figure 1A). We used CRISPR/Cas9-mediated genome editing to knock in the p.Ile772Met mutation. In the resulting offspring, the p.Ile772Met mutation was identified by Sanger sequencing of genomic DNA (Figure 1B). We confirmed expression of the mutant allele by sequencing adrenal cDNA (Figure 1C). We also obtained mice with a heterozygous 1 bp deletion, leading to the generation of a stop codon (p.Leu783*, *Cacna1d^+/–^*, data not shown). However, when backcrossing *Cacna1d^+/–^* mice, no *Cacna1d^–/–^* mice were found among 147 offspring genotyped at weaning (probability of 4.3 × 10^–19^ with 25% homozygous offspring expected; 46 *Cacna1d^+/+^* and 101 *Cacna1d^+/–^* mice found). Genotyping of 9 embryos at stage E8.5 revealed 1 *Cacna1d^–/–^* mouse, suggesting premature mortality at later embryonic stages or early postpartum.

### Gross phenotype, survival, and breeding of Cacna1d^Ile772Met/+^ mice.

**Figure 2:**
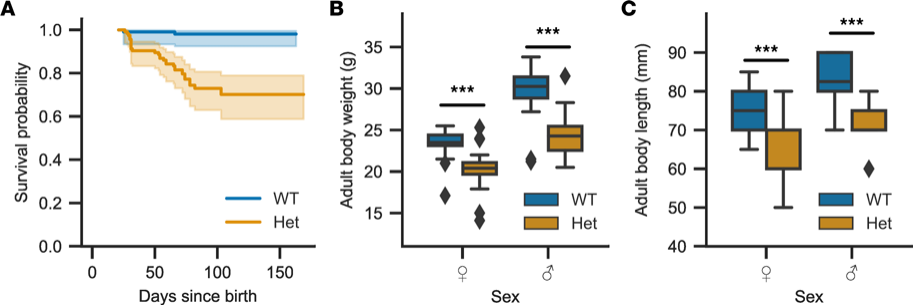
*Cacna1d*^Ile772Met/+^ mice show higher mortality and gain less weight than WT mice. (**A**) Kaplan-Meier curve showing survival probability of *Cacna1d^Ile772Met/+^* (Het, orange) and WT (blue) mice relative to the number of mice weaned at 21 days. The 95% CI is plotted in a lighter shade around the mean. Mortality is higher in *Cacna1d^Ile772Met/+^* than in WT mice from weaning to approximately 100 days of age (log rank test; *n*_WT_ = 103, *n*_Het_ = 114; *P* < 0.005; χ^2^= 24.39, df = 1). (**B**) Body weight determined during the SHIRPA assessment prior to behavioral phenotyping experiments (age 13–18 weeks). *Cacna1d^Ile772Met/+^* mice are lighter than WT littermates regardless of sex (Mann-Whitney *U*; *n*_WT,_ _female_ = 26, *n*_Het,_ _female_ = 18, *P* = 0.0004, *U* = 329.5; *n*_WT,_ _male_ = 22, *n*_Het,_ _male_ = 20, *P* = 6.2×10^–5^, *U* = 333). (**C**) Body length measured during the SHIRPA assessment show that *Cacna1d^Ile772Met/+^* mice are shorter than WT littermates regardless of sex (Mann-Whitney *U*; *n*_WT,_ _female_ = 22, *n*_Het,_ _female_ = 17, *P* = 7.0×10^–5^, *U* = 56; *n*_WT,_ _male_ = 18, *n*_Het,_ _male_ = 17, *P* = 2.3×10^–5^, *U* = 33). ***P < 0.001.

We initially attempted to propagate *Cacna1d^Ile772Met/+^* mice by breeding them with C57BL/6J WT mice. Despite multiple attempts, no pregnancies were achieved when housing male *Cacna1d^Ile772Met/+^* mice with female WT mice, and no plugs were observed. Video recordings demonstrated attempted mountings; however, male *Cacna1d^Ile772Met/+^* mice appeared to lack the strength and/or motor coordination for successful mating (Supplemental Table 1 and Supplemental Videos 1–4; supplemental material available online with this article; https://doi.org/10.1172/jci.insight.162468DS1; observation of 3 male WT and 3 male *Cacna1d^Ile772Met/+^* mice). Breeding of female *Cacna1d^Ile772Met/+^* mice with male WT mice resulted in pregnancies; however, the offspring were found dead shortly after birth. Sperm isolated from a *Cacna1d^Ile772Met/+^* mouse showed normal motility and was used for in vitro fertilization (IVF), which resulted in the birth of viable offspring. In the second generation, conventional breeding was again unsuccessful, and all further experimental animals were generated by IVF. Among animals genotyped at weaning, *Cacna1d^Ile772Met/+^* mice (*n* = 79) were not underrepresented compared with WT (*n* = 68), suggesting that perinatal lethality was not increased. However, we observed increased mortality of *Cacna1d^Ile772Met/+^* mice between weaning and approximately 100 days of age (Figure 2A), after which no further lethality was observed. Pathological investigation in one case revealed brownish visceral organs and a sparsely filled gastrointestinal tract, but no specific cause of death was determined. Both male and female *Cacna1d^Ile772Met/+^* mice showed significantly reduced body weight and shorter body length compared with WT littermates (Figure 2, B and C). Organ weights of WT male mice were significantly heavier with the notable exception of brain, with less pronounced effects in female mice (Supplemental Table 2). We next investigated *Cacna1d^Ile772Met/+^* mice for features of PASNA syndrome.

### Cacna1d^Ile772Met/+^ mice have elevated aldosterone/renin ratios as a sign of PA.

**Figure 3:**
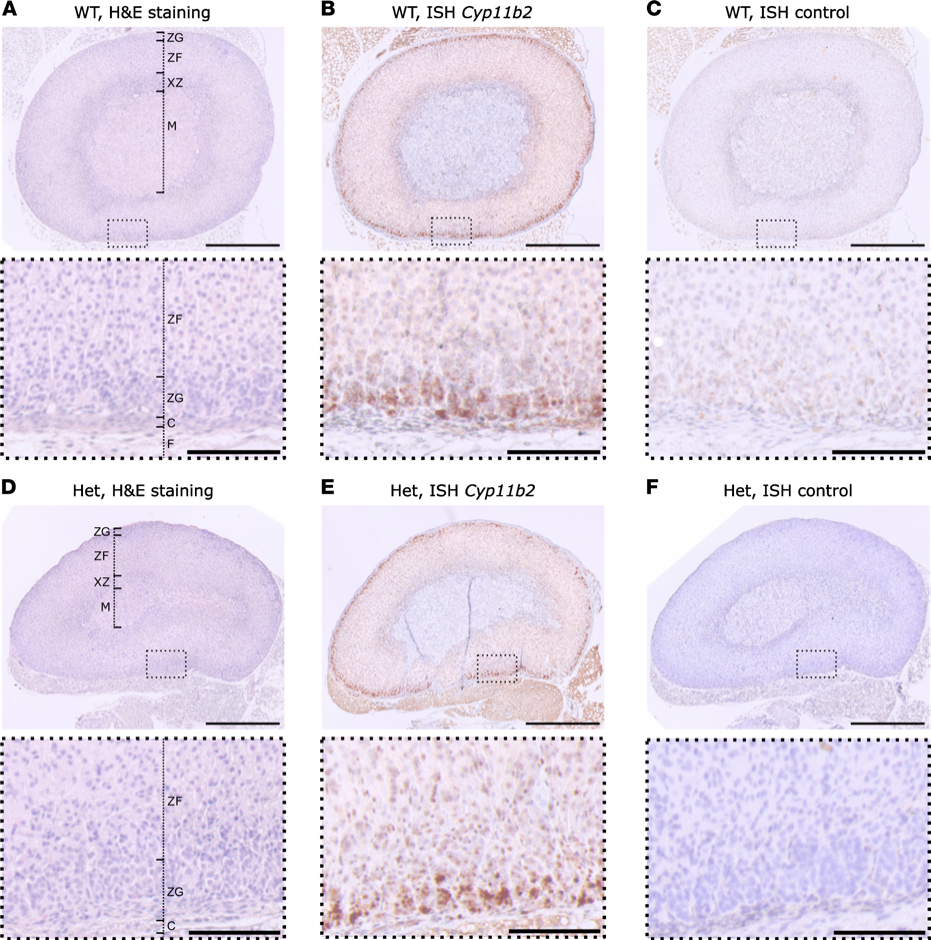
Adrenal histology and *Cyp11b2* ISH of *Cacna1d^Ile772Met/+^* and WT. (**A** and **D**) H&E staining of FFPE adrenal sections from 14-week-old female WT (**A**) or *Cacna1d*^Ile772Met/+^ mice (Het, **D**). F, fat; C, capsule; ZG, zona glomerulosa; ZF, zona fasciculata; XZ, x-zone, M, medulla. (**B** and **E**) ISH of WT or Het mice. A probe for the detection of *Cyp11b2* mRNA was used. Staining is confined to the ZG. (**C** and **F**) Controls for ISH without any probes are shown. The stainings shown in this figure are representative for 8 WT (6 female, 2 male) and 6 Het (3 female, 3 male). Scale bars: 500 μm (overview, top) or 100 μm (magnification, bottom).

**Figure 4:**
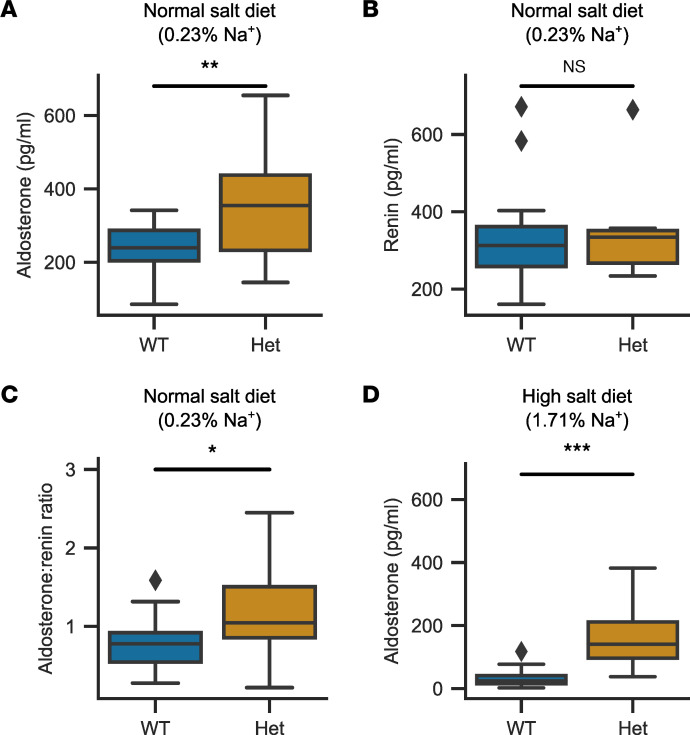
Serum aldosterone and aldosterone/renin ratio are increased in 18-week-old *Cacna1d*^Ile772Met/+^ mice. (**A**) Serum aldosterone levels as determined by ELISA are elevated in *Cacna1d^Ile772Met/+^* (Het) versus WT mice (2-tailed *t* test; *n*_WT_ = 19, *n*_Het_=12, *P* = 0.005, *t* = –3.06). (**B**) Renin concentrations as determined by ELISA exhibit no significant difference between WT and Het mice (Mann-Whitney *U* test; *n*_WT_ = 19, *n*_Het_ = 13, *P* = 0.94, *U* = 121). (**C**) The aldosterone/renin ratio is increased in Het mice (2-tailed *t* test; *n*_WT_ = 19, *n*_Het_=12, *P* = 0.02, *t* = –2.47). (**D**) A 2-week high-salt diet (4% Na^+^ in chow, 1% NaCl in drinking water) resulted in lower aldosterone levels in both genotypes. However, Het mice still exhibited significantly higher serum aldosterone levels (Mann-Whitney *U* test; *n*_WT_ = 31, *n*_Het_ = 28, *P* = 1.21 × 10^–9^, *U* = 33). *P < 0.05, **P < 0.01, ***P < 0.001.

We assessed the morphology of WT and mutant adrenal glands by H&E staining and ISH for *Cyp11b2* (encoding aldosterone synthase as a marker of the aldosterone-producing zona glomerulosa [ZG]). No changes in zonation were seen, and the zona glomerulosa was not enlarged (Figure 3) (ZG thickness; WT: *n* = 8, 24.8 ± 9.1 μm, *Cacna1d^Ile772Met/+^* (Het): *n* = 6: 19.9 ± 6.9 μm; Mann-Whitney *U* test: *U* = 32, *P* = 0.34). Serum aldosterone levels were significantly elevated in *Cacna1d^Ile772Met/+^* mice compared with WT (Figure 4A) (WT: *n* = 19, 233.7 ± 67.2 pg/mL; Het: *n* = 12, 360.3 ± 160.3 pg/mL), but renin concentration was not different (Figure 4B) (WT: *n* = 19, 330.1 ± 126.9 pg/mL; Het: *n* = 13, 333.5 ± 108.5 pg/mL). This resulted in a significantly elevated aldosterone/renin ratio in *Cacna1d^Ile772Met/+^* mice, indicative of PA (Figure 4C) (WT: *n* = 19, 0.78 ± 0.32; Het: *n* = 12, 1.17 ± 0.58). However, adrenal *Cyp11b2* expression was not significantly elevated as determined by quantitative PCR (qPCR) (Supplemental Figure 1A). We did not observe major electrolyte abnormalities in *Cacna1d^Ile772Met/+^* mice (Table 1).

### Aldosterone remains elevated on a high-salt diet in Cacna1d^Ile772Met/+^ mice.

To assess whether aldosterone production is — at least partially — uncoupled from regulation by the renin-angiotensin system in *Cacna1d^Ile772Met/+^* mice, we fed mice a high-salt diet (4% NaCl in food, 1% NaCl in drinking water) for 2 weeks and measured aldosterone at the end of this period. As expected, aldosterone levels strongly declined in WT mice (32.8 ± 26.6 pg/mL). In *Cacna1d^Ile772Met/+^* mice, levels declined slightly but remained much higher than in WT (Figure 4D; 156.3 ± 83.3 pg/mL). Similarly, adrenal *Cyp11b2* levels were significantly elevated in heterozygous mice compared with WT mice after a high-salt diet (Supplemental Figure 1C). Renin concentrations could not be reliably assessed due to the use of isoflurane for anesthesia (30) to prevent induction of seizures (see below), but renal renin expression did not significantly differ between WT and mutant mice after a high-salt diet (Supplemental Figure 1D).

### Cacna1d^Ile772Met/+^ mice show no signs of hyperinsulinemia or hypoglycemia.

Serum glucose levels at euthanasia were similar in WT and *Cacna1d^Ile772Met/+^* mice (Table 1). Because isoflurane anesthesia (see below) can increase blood glucose levels but does not affect insulin release (31), we additionally determined serum insulin levels by ELISA. We did not observe significant differences between WT and *Cacna1d^Ile772Met/+^* mice (Supplemental Figure 2).

### Cacna1d^Ile772Met/+^ mice show elevated zona glomerulosa calcium levels.

**Figure 5:**
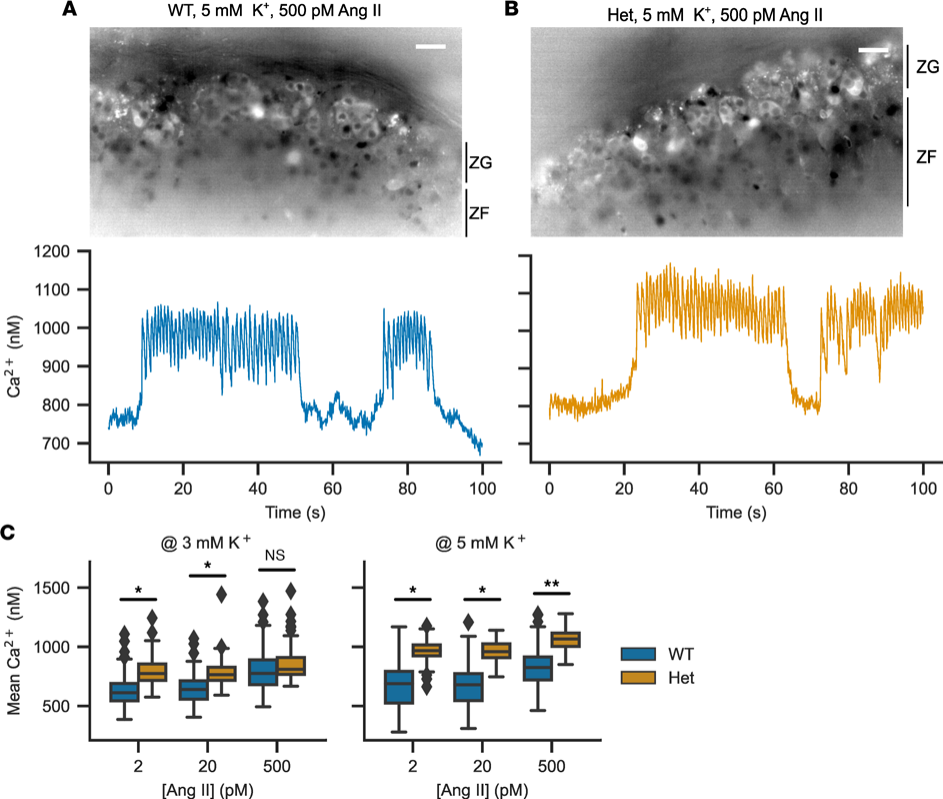
Calcium imaging in acute adrenal slices to determine the response of the zona glomerulosa to potassium and angiotensin II. (**A** and **B**) Representative image of a WT (**A**) or Ile772Met/+ (**B**) adrenal slice stained with Fura-2 AM (top). On the bottom, representative traces are shown. Scale bars: 10 μm. (**C**) Mean calcium concentrations in zona glomerulosa cells determined by imaging of Fura-2 AM–stained slices. The extracellular concentration of angiotensin II was varied between 2 and 500 pM in the presence of either 3 (left) or 5 mM K^+^ (right) (likelihood ratio test of linear mixed models; exact statistical values are given in Table 2; *P <0.05, **P < 0.01).

We performed calcium imaging of adrenal slices stained with Fura-2 AM (ratiometric dye, allowing for determination of absolute calcium concentrations). We used variable extracellular concentrations of K^+^ and angiotensin II (Ang II) to model effects of the 2 main stimuli of aldosterone production. The pattern of calcium oscillations was largely unaffected (Figure 5, A and B). *Cacna1d^Ile772Met/+^* mice showed significantly elevated intracellular calcium levels compared with WT across almost all concentrations of Ang II and extracellular K^+^. They showed the expected increase in calcium signaling in response to increased extracellular K^+^ and supraphysiologic concentration of Ang II (Figure 5C and Table 2). At a concentration of 2 pM Ang II, cells only rarely exhibited calcium spikes, the observed increase in mean calcium therefore reflects an increase in baseline calcium levels (Figure 5C). The higher intracellular calcium levels were not associated with increased frequencies of calcium spiking (Supplemental Figure 3A), and bursting parameters were similar, apart from increased numbers of bursts under some conditions (Supplemental Figure 3B).

### Anesthesia with ketamine and xylazine induces tonic-clonic seizures in Cacna1d^Ile772Met/+^ mice.

We did not observe spontaneous seizure activity in *Cacna1d^Ile772Met/+^* mice in video recordings of 4 mice spanning approximately 24 hours each. However, upon i.p. injection of ketamine and xylazine for anesthesia, 10 of 13 *Cacna1d^Ile772Met/+^* mice showed abnormal jerking movements and tonic-clonic seizures (Supplemental Video 5). No seizures were observed in 19 similarly treated WT mice (*P* < 1 × 10^–5^, Fisher’s exact test). We subsequently changed anesthesia to isoflurane, under which we did not observe further seizures.

### Cacna1d^Ile772Met/+^ mice show multiple neurologic abnormalities.

**Figure 6:**
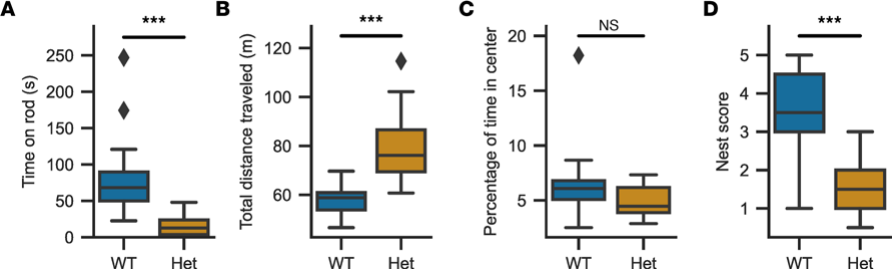
*Cacna1d*^Ile772Met/+^ mice show motor abnormalities as well as deficits in nest construction. (**A**) WT mice stay significantly longer on the rotating rod in rotarod experiments than *Cacna1d^Ile772Met/+^* (Het) mice (Mann-Whitney *U*; *n*_WT_ = 19, *n*_Het_ = 19, *P* = 3.48 × 10^–12^, *U* = 2852.5). (**B**) The total distance covered by Het mice in 10 minutes in the open-field experiment is significantly higher than for WT as a sign of hyperlocomotion (2-tailed *t* test; *n*_WT_ = 19, *n*_Het_ = 19, *P* = 2.6 × 10^–7^, *t* = -6.32). (**C**) The percentage of the open-field observation time spent in the center of the box is not different between genotypes (Mann-Whitney *U*; *n*_WT_ = 19, *n*_Het_ = 19, *P* = 0.08, *U* = 119). (**D**) Het mice are largely unable to build well-formed nests over 18 hours, as indicated by the lower nest scores (Mann-Whitney *U*; *n*_WT_ = 19, *n*_Het_ = 19, *P* = 0.0006, *U* = 281.5). ***P < 0.001.

**Figure 7:**
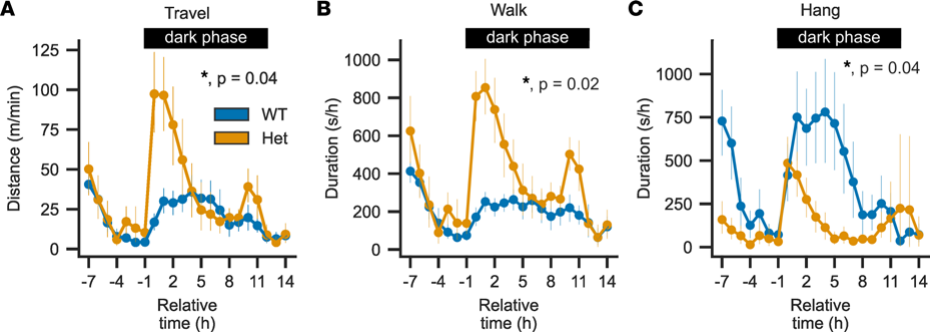
*Cacna1d*^Ile772Met/+^ mice show hyperlocomotion in response to changes in ambient light but are unable to maintain a hanging position at the top of the cage. (**A**–**C**) Course of 3 groups of home-cage behaviors over time for 24 hours (see Supplemental Figure 5 for all other groups). *Cacna1d*^Ile772Met/+^ mice show hyperlocomotion as indicated by longer distances traveled (**A**; likelihood ratio test of linear mixed models; *n*_WT_ = 19, *n*_Het_ = 17; *P*_adj_ = 0.02, *z* = 0.085) together with increased periods spent moving around (**B**; likelihood ratio test of linear mixed models; *n*_WT_ = 19, *n*_Het_ = 17; *P*_adj_ = 0.02, *z* = 0.00208). Hanging from the lid of the cage is significantly reduced in *Cacna1d*^Ile772Met/+^ mice compared with WT (**C**; likelihood ratio test of linear mixed models; *n*_WT_ = 19, *n*_Het_ = 17; *P*_adj_ = 0.04, *z* = 2.631). For all statistical models, genotype was used as fixed and recording as well as time as random factors. All *P* values are adjusted for 10 comparisons (including the remaining behaviors shown in Supplemental Figure 5) using the FDR method. Data are shown as mean ± 95% CI.

We performed a modified SmithKline Beecham, Harwell, Imperial College, and Royal London Hospital phenotype assessment (SHIRPA) test battery as a generalized neurological screen of WT and heterozygous mice (Supplemental Table 3) (32). Besides reduced body weight (Figure 2B), this revealed tremor almost exclusively in *Cacna1d^Ile772Met/+^* mice (36 of 37 in Het versus 2 of 42 in WT; *P* = 1.9 × 10^–19^, Fisher’s exact test) and reduced grip strength and grasping (wire maneuver test; *n*_WT_ = 42, *n*_Het_ = 37; median score WT: 0, median Het: 2; Mann-Whitney *U*, *U* = 523, *P* = 3.7 × 10^–10^). Auditory startle response to a tone from a clicker was intact in both WT and *Cacna1d^Ile772Met/+^* mice. We also did not find evidence of impaired olfaction in a buried food-seeking test (33) (Supplemental Figure 4A), and mice showed normal recognition memory of a familiar object (Supplemental Figure 4B). To further test motor function, we performed the rotarod test. *Cacna1d^Ile772Met/+^* mice showed impaired coordination, with reduced times spent on the rod (Figure 6A) (WT: *n* = 19, 80.8 ± 66.0 s; Het: *n* = 19, 15.3 ± 20.0 s). In the open-field test, *Cacna1d^Ile772Met/+^* mice exhibited hyperlocomotion (Figure 6B) (WT: *n* = 19, 57.8 ± 5.6 m/10 minutes; Het: *n* = 19, 79.6 ± 14.0 m/10 minutes) without signs of increased anxiety (similar times spent in the center of the box versus near the walls; Figure 6C) (WT: *n* = 19, 6.4% ± 5.6%; Het: *n* = 19, 4.9% ± 3.7%). *Cacna1d^Ile772Met/+^* mice showed severely impaired nest building scores (Figure 6D; mean ± SD; WT: *n* = 19, 2.7 ± 1.3; Het: *n* = 19, 1.3 ± 0.5), which have been linked to structural or functional brain deficits (34). To further investigate these abnormalities, we performed home cage screening (HCS), in which behavior is automatically categorized based on video recordings of singly housed mice in cages for a period of 24 hours (Supplemental Figure 5). *Cacna1d^Ile772Met/+^* mice showed increased locomotion (Figure 7, A and B) compared with WT mice particularly at the start of the dark period (active period of mice), with decreasing locomotion in the later phase of the dark period. A similar peak in activity was observed after the change to the light phase (Figures 7, A and B). This was not due to a change in resting patterns (Supplemental Figure 5). In line with reduced grip strength observed in the SHIRPA test, *Cacna1d^Ile772Met/+^* mice spent less time hanging at the top of the cage lid (Figure 7C).

### Social behavior in Cacna1d^Ile772Met/+^ mice.

**Figure 8:**
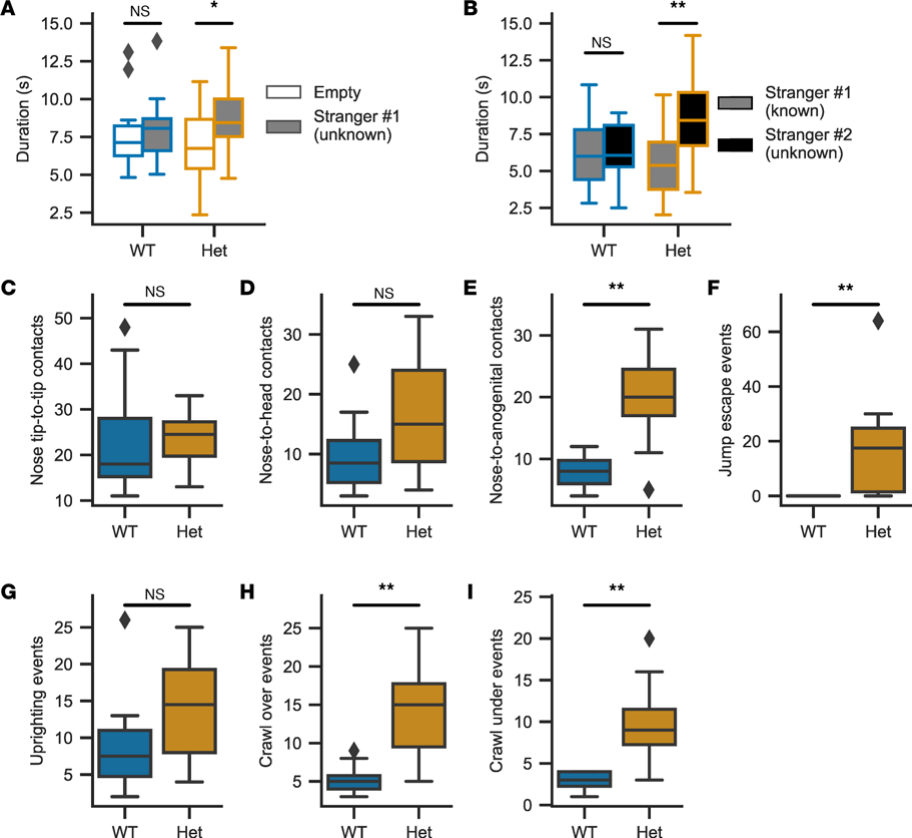
*Cacna1d*^Ile772Met/+^ mice tolerate free interaction but show avoidance behavior in forced interactions. (**A**) In the second session of the 3-chamber test, *Cacna1d*^Ile772Met/+^ (Het) mice show a slight preference for social interaction as seen by longer total durations spent near the unfamiliar stranger (Mann-Whitney *U* test; *n*_WT_ = 19, *P* = 0.22, *U* = 201; *n*_Het_ = 18, *P* = 0.04, *U* = 251). (**B**) During the third session of the 3-chamber test, *Cacna1d ^Ile772Met/+^* — but not WT mice — show a preference for the unfamiliar stranger (Mann-Whitney *U* test; *n*_WT_ = 19, *P* = 0.60, *U* = 162; *n*_Het_ = 18, *P* = 0.001, *U* = 70) (**C**–**I**) Parameters determined in the social proximity test. The increase in nose-to-anogenital, crawl-over, crawl-under, and jump escape events are considered indicative of increased anxiety and avoidance behavior (Mann-Whitney *U* test; *n*_WT_ = 10, *n*_Het_ = 8). *P* = 0.66, *U* = 34.5 (**C**); *P* = 0.17, *U* = 24 (**D**); *P* = 0.008, *U* = 9.5 (**E**);*P* = 0.002, *U* = 10 (**F**); *P* = 0.1, *U* = 21 (**G**); *P* = 0.003, *U* = 6 (**H**); *P* = 0.002, *U* = 5.5 (**I**). *P < 0.05, **P < 0.01.

We used the 3-chamber social test to detect changes in preference for a social stimulus (unknown mouse) compared with an empty cage. *Cacna1d^Ile772Met/+^* mice showed a marked preference for a novel social stimulus over a novel empty object (small grid cage) (Figure 8A). Similarly, *Cacna1d^Ile772Met/+^* mice again preferred a novel social stimulus (new unknown mouse) over a known social stimulus (Figure 8B), whereas no significant preference was seen in WT littermates. Exploration of the novel social stimulus is mostly limited to olfaction, manifesting as sniffing. Counting the number of sniffing events at the new unknown mouse relative to the known mouse revealed a similar pattern with no difference for WT (*n* = 19, known: 28 ± 9 sniffs, unknown: 29 ± 7 sniffs, Mann-Whitney *U*, *U* = 146, *P* = 0.32) but a preference for the novel stimulus in *Cacna1d^Ile772Met/+^* mice (*n* = 19, known: 36 ± 10 sniffs, unknown: 55 ± 14 sniffs, Mann-Whitney *U*, *U* = 51, *P* = 2 × 10^–4^). We additionally performed a social proximity test (forced social interaction in a constrained space) to investigate whether *Cacna1d^Ile772Met/+^* mice show evidence of autistic features. Compared with WT mice, *Cacna1d^Ile772Met/+^* mice showed significantly more nose-to-anogenital contacts, jump escape events, uprighting events, crawl-over events, and crawl under-events when in contact with an unknown mouse, but there was no significant increase in nose tip–to–nose tip contacts or nose-to-head contacts (Figure 8, C–I).

### Cacna1d^Ile772Met/+^ mice do not show evidence of structural brain abnormalities.

To investigate whether the observed neurological abnormalities were due to structural brain abnormalities, we assessed overall brain morphology using Nissl staining (35) (Supplemental Figure 6). No major abnormalities were detected, and structures such as cortex, hippocampus, midbrain, cerebellum, thalamus, hypothalamus, and striatum appeared unaltered. Analysis of cell density and nucleus size in the striatum, which plays central roles in not only movement planning and execution but also social behavior (36), did not reveal significant differences between the 2 genotypes. Similarly, the pattern of dopaminergic neurons in the midbrain (including the substantia nigra, crucial for voluntary movement; ref. 37) and the expression of the rate-limiting enzyme in dopamine synthesis, tyrosine hydroxylase (*Th*), appeared unchanged (Supplemental Figure 7).

### Incubation with isradipine lowers intracellular calcium levels in zona glomerulosa cells.

**Figure 9:**
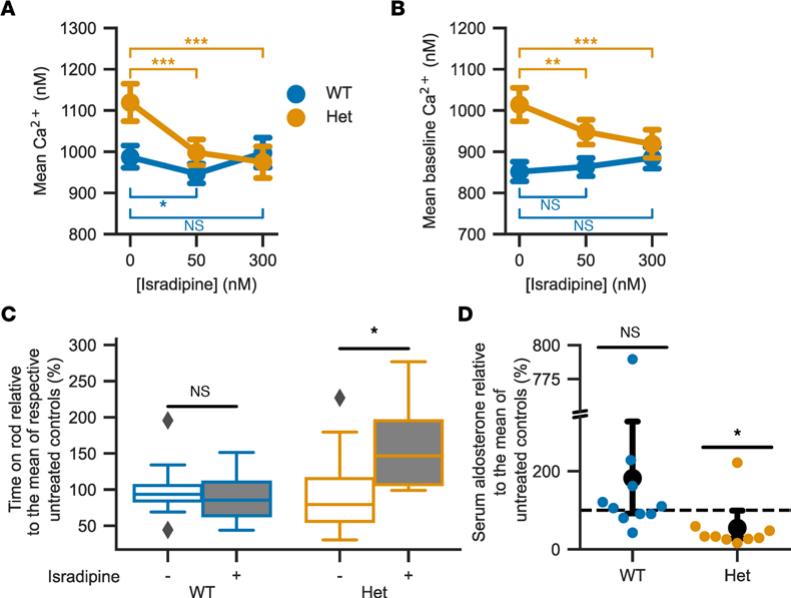
Treatment with the L-type calcium channel blocker isradipine partially reverses the effects of the Ile772Met mutation. (**A**) Isradipine lowers mean intracellular calcium concentrations (likelihood ratio test of linear mixed models; 50 nM versus 0 nM isradipine: *n*_WT,_ _cells_ = 143, *n*_WT,_ _slices_ = 12, *P*_adj_ = 0.028, χ^2^ = 6.08, df = 1; *n*_Het,_ _cells_ = 85, *n*_Het,_ _slices_ = 9, *P*_adj_ = 2.16 × 10^–7^, χ^2^(1) = 28.36; 300 nM versus 0 nM isradipine: *n*_WT,_ _cells_ = 143, *n*_WT,_ _slices_ = 12, *P*_adj_ = 1.0, χ^2^(1) = 0.22; *n*_Het,_ _cells_ = 85, *n*_Het,_ _slices_ = 9, *P*_adj_ = 2.56 × 10^–7^, χ^2^(1) = 27.89; 4 mM K^+^ and 100 pM Ang II). (**B**) Isradipine lowers baseline intracellular calcium concentrations in Het mice (cells/slices from **A**; mean ± 95% CI; likelihood ratio test of linear mixed models; 50 nM isradipine: WT: *P*_adj_ = 0.8, χ^2^(1) = 0.72; Het:, *P*_adj_ = 0.0016, χ^2^(1) = 11.15; 300 nM isradipine: WT: *P*_adj_ = 0.11, χ^2^(1) = 3.72; Het: *P*_adj_ = 4.48×10^–8^, χ^2^(1) = 32.82). *P* values in **A** and **B** were adjusted for multiple comparisons using the Bonferroni procedure. (**C**) Four hours after the last isradipine dose, treated (+) Het show a significant improvement in rotarod performance compared with untreated controls (–) (2-tailed *t* test; *n*_WT,_ _treated_ = 10, *n*_WT,_ _control_ = 11, *P*_wt_ = 0.63, *t*_wt_ = -0.50; *n*_Het,_ _treated_ = 9, *n*_Het,_ _control_ =9, *P*_Het_ = 0.03, *t*_Het_ = 2.17). Values were normalized to the mean of untreated mice of the same genotype. (**D**) In Het mice, serum aldosterone is lower relative to controls (95% CI, 92.1–327.1, Het: 27.7–99.0; mean ± 95% CI, black circles ± whiskers; 100% of controls is indicated with dashed line, and individual values are shown as colored circles). The break in the *y* axis extends from 340 to 750%. See Supplemental Figure 8A for absolute values.

To assess whether increased intracellular zona glomerulosa calcium is sensitive to inhibition by a clinically approved drug, we incubated slices with the dihydropyridine-class calcium channel blocker isradipine. We used a concentration of 50 nM, which can be reached in mouse plasma following s.c. delivery with osmotic minipumps (28), as well as 300 nM, which, in a heterologous expression system, inhibited virtually all Ca_V_1.3 channels (38). Both WT and *Cacna1d^Ile772Met/+^* glomerulosa cells responded with a decrease in mean intracellular Ca^2+^, and levels in treated *Cacna1d^Ile772Met/+^* cells were lowered to approximately the levels seen in untreated WT cells (Figure 9A and Table 3). Notably, isradipine lowered baseline Ca^2+^ concentrations exclusively in *Cacna1d^Ile772Met/+^* cells (Figure 9B and Table 3).

### Cacna1d^Ile772Met/+^ mice respond to oral therapy with isradipine.

Implantation of osmotic minipumps (28) is a stressful invasive procedure. Given the already-increased mortality in *Cacna1d^Ile772Met/+^* mice, we instead chose oral administration of isradipine. Slow-release isradipine is approved for the therapy of hypertension and taken once daily in humans (39). We fed *Cacna1d^Ile772Met/+^* mice and WT controls once daily with 12.5 mg/kg of a slow-release isradipine formulation in sweetened yogurt. Both the treatment and the placebo group (sweetened yogurt only) typically rapidly and voluntarily ingested the offered yogurt; mice that did not ingest the yogurt were excluded from further analysis. After a 2-week treatment phase, we continued treatment while performing behavioral testing to assess effects on neurologic behavior. We performed rotarod testing approximately 4 hours after yogurt or isradipine feeding, at a time when peak plasma levels are reached in humans (40). In WT animals, no difference in rotarod performance in the therapy group versus placebo was observed. Conversely, the time spent on the rod was, on average, 68% higher in *Cacna1d^Ile772Met/+^* mice treated with isradipine than in *Cacna1d^Ile772Met/+^* mice that received placebo (Figure 9C), even though absolute performance still did not reach WT levels (Table 4). No effect was seen in the open-field test 18 hours after the last dose (Supplemental Figure 8B), at which time we expected trough plasma levels (40). Similarly, no change in nest building performance was seen (test covered peak as well as trough plasma levels of isradipine) (Supplemental Figure 8C). We assessed aldosterone levels from a terminal blood collection approximately 20 hours after the last dose. Treatment led to a reduction of serum aldosterone relative to untreated controls in *Cacna1d^Ile772Met/+^* but not WT mice (Figure 9D). Renin concentrations could not be reliably assessed due to the use of isoflurane for anesthesia (30) to prevent induction of seizures (see above).

## Discussion

The *Cacna1d^Ile772Met/+^* mice we here generated closely model human PASNA syndrome, showing PA, increased seizure susceptibility, and neurologic abnormalities, including motor deficits and slight social impairment. *Cacna1d^Ile772Met/+^* mice had high aldosterone levels that remained elevated (along with elevated *Cyp11b2* levels) after a high-salt diet, confirming PA (41). Mechanistically, calcium concentrations in glomerulosa cells of *Cacna1d^Ile772Met/+^* mice were elevated, but the frequency of spiking and bursting parameters were mostly unchanged (Figure 5 and Supplemental Figure 3). Baseline calcium levels were elevated in the virtual absence of spiking (during perfusion of 2 pM of Ang II or during pauses of spiking at stronger stimulation (Figure 5), consistent with calcium permeability of the mutant channel at hyperpolarized potentials. Like other models of mild PA (42–44), *Cacna1d*–knockin mice had unsuppressed renin. Stress-induced stimulation of renin release during application of anesthesia and blood draw may act as a confounder (45). Introduction of the germline Ile772Met mutation in mice did not cause macroscopic or microscopic glomerulosa hyperplasia at 14 weeks of age. Similarly, the child with PASNA syndrome in whom the corresponding Ile770Met de novo germline mutation was identified did not show macroscopic adrenal abnormalities by computed tomography (8). Given that the corresponding Ile770Met mutation has been identified in human aldosterone-producing adenomas (8), factors other than somatic *CACNA1D* mutations may be required for increased cell mass in human tumors (46). Because knockin mice were fragile and showed increased mortality, we could not perform invasive blood pressure monitoring in *Cacna1d^Ile772Met/+^* mice. Other models with mild PA show moderately elevated blood pressure (42, 43, 47). Hypoglycemic hyperinsulinism was not reported in the child with germline Ile770Met mutation (8). Likewise, we did not detect hyperinsulinemic hypoglycemia in our mice. However, we cannot exclude transient neonatal hypoglycemia (8, 48).

The requirement for IVF limited the scope of our studies. Video recordings demonstrated several attempts of *Cacna1d^Ile772Met/+^* males to mount females. However, these remained unsuccessful, likely due to their impaired neuromotor phenotype (Supplemental Table 1). We suspect that the offspring of female *Cacna1d^Ile772Met/+^* mice did not survive due to lack of maternal care and/or feeding because animals carried by synchronized WT recipients survived.

The overall neurologic phenotype of *Cacna1d^Ile772Met/+^* mice is likely due to functional alterations rather than structural brain abnormalities (Supplemental Figure 6). Ca_V_1.3 channels play an important role in the dopaminergic system (49). Ca_V_1.3 is expressed in substantia nigra dopamine neurons that project to the dorsal striatum and are important for voluntary movements (50). Ca^2+^ entry via Ca_V_1 channels into these neurons can be inhibited by isradipine, leading to a reduction in average and peak intracellular [Ca^2+^] levels (51). Thus, increased calcium and subsequent dopamine signaling may explain some aspects of the neurological phenotype of *Cacna1d^Ile772Met/+^* mice. Accordingly, functional changes in dopaminergic neurons of the medial substantia nigra and GABAergic medium spiny neurons in the dorsal striatum were found in a related mouse model with gain-of-function *Cacna1d* mutation (52).

How anesthesia with ketamine and xylazine induced seizures remains to be determined. Very few cases of ketamine-induced seizures have been described in humans and in cats (53), and a case of a potentially xylazine-induced seizure in a horse has been reported (54). It is conceivable that spontaneous seizures also occur in *Cacna1d^Ile772Met/+^* mice, and such events could contribute to the increased lethality of the strain. Video recordings over 24 hours did not show such events, but they were performed at age 17–18 weeks, when lethality was no longer increased compared with WT.

Regarding social interactions, we unexpectedly observed an increased preference of *Cacna1d^Ile772Met/+^* mice for unknown mice in the 3-chamber social preference assay (Figure 8). While the absence of the expected preference for unknown mice in WT mice may point to suboptimal assay conditions, general social interaction and recognition of unknown mice is likely not impaired in *Cacna1d^Ile772Met/+^* mice. We note that different versions of this assay have been developed, with discrepancies in phenotypic descriptions of several autism spectrum disorder mouse models (55). Increased nose-to-anogenital, crawl-under, and crawl-over behaviors of *Cacna1d^Ile772Met/+^* mice in the social proximity assay may reflect avoidance of reciprocal frontal orientations, analogous to gaze aversion in humans with autism spectrum disorder (56). Crawl-under behavior and jump escape have been associated with anxiety and defensiveness (56). Hyperlocomotion, observed in open-field and HCS, with resulting increased overall interactions, may override decreased nose tip–to–nose tip behavior that is otherwise characteristic of autism-like behavior in mice (56). Autism-like behavior is not a predominant feature in *Cacna1d^Ile772Met/+^* mice. This feature appears to be more prominent in mice homozygous for a *Cacna1d* mutation associated with autism in humans (52), demonstrating that the spectrum from disorder to disease associated with different mutations in humans is well reflected in mouse models. Olfaction and audition, which could have affected social behavior, appeared largely unaffected in *Cacna1d^Ile772Met/+^* mice (Supplemental Table 3 and Supplemental Figure 4A). In the SHIRPA test, there were no major neuromotor abnormalities other than impaired grasping in the wire maneuver and tremor (Supplemental Table 3), yet nest construction was severely impaired (Figure 6D).

Interestingly, contrary to a published *Cacna1d*-KO model with deafness, bradycardia (57), and impaired motor performance (58), our *Cacna1d^–/–^* mice were not viable. It would be interesting to determine whether alternative splicing (several splice isoforms, including a long and a short isoform, have been reported; ref. 59) or other mechanisms enable residual Ca_V_1.3 function in the published model.

The most remarkable result of our study is the partial response of PASNA syndrome features to therapy with isradipine. Only knockin mice showed improved rotarod performance 4 hours after isradipine administration, when peak plasma levels are expected. Furthermore, in knockin mice, intracellular calcium levels in adrenal slices and plasma aldosterone levels declined upon isradipine administration. When analyzing baseline calcium concentrations outside of spiking events, the effect of isradipine was strikingly restricted to *Cacna1d^Ile772Met/+^* mice (Figure 9B), suggesting that published shifts of channel activation and inactivation to more hyperpolarized potentials (8) cause abnormal constitutive calcium influx at or close to the glomerulosa resting membrane potential in *Cacna1d^Ile772Met/+^* mice. This may explain why isradipine reduced aldosterone levels in knockin mice. Given that isradipine is approved for the therapy of hypertension, calcium channel blocker therapy could be a treatment option in patients with PA due to somatic *CACNA1D* mutations. Isradipine appears particularly suited because it has previously been shown to be one of the dihydropyridines with the greatest affinity toward Ca_V_1.3 (28, 29). Different Ca_V_1.3 mutations vary in their response to calcium channel inhibitors, and it would be interesting to correlate clinical responses with the sensitivity of variants identified by sequencing after adrenalectomy (17). New blockers that are more specific to Ca_V_1.3 could have even more pronounced effects (60). The noninvasive identification of patients with *CACNA1D* mutations is challenging given the requirement for tissue from the lesion for genotyping. One approach may be the exclusion of the most prevalent *KCNJ5* mutations by steroid profiling (61), followed by a trial of calcium channel blockers. Our data also suggest that therapeutic trials of calcium channel inhibitors could be promising in patients with PASNA syndrome. These patients often have debilitating neurological impairment, and even minor improvements could be clinically relevant. Use of amlodipine and nifedipine, respectively, has been described in 2 cases (8, 48), but few details were provided, and these substances show only low affinity toward Ca_V_1.3 (29). Carefully designed protocols (perhaps using isradipine, which can be administered in yogurt to patients with difficulty swallowing) and detailed documentation could be a step forward, perhaps followed by the development of more specific Ca_V_1.3 inhibitors (60, 62).

## Methods

### Generation and propagation of the Cacna1d^I772M/+^ mouse model.

CRISPR/Cas9-mediated mutagenesis in the *Cacna1d* gene (Ile772Met) was performed at the Yale Genome Editing Center as described previously (42) using fertilized eggs from C57BL/6J mice. See Supplemental Methods for more information.

### High-salt diet.

We fed mice with food pellets containing 1.71% Na^+^ (4% NaCl; EF15431-347, ssniff Spezialdiäten) ad libitum for 2 weeks. Drinking water was supplemented with 1% NaCl (w/v) with ad libitum access. Mice were habituated to handling during these 2 weeks. Afterward, samples were collected as described below.

### IVF.

IVF was performed at the Yale Genome Editing Center and the Transgenic Technologies Core Facility within the Forschungseinrichtungen für Experimentelle Medizin (FEM, Charité - Universitätsmedizin Berlin) using C57BL/6J females as egg donors and sperm from male *Cacna1d*^I772M/+^ mice. Sperm harvest and embryo transfer were performed as published (63). See Supplemental Methods for more information.

### Adrenal calcium imaging.

For acute slice preparations, adrenal glands were extracted from 10- to 18-week-old mice (WT: 7 female and 9 males; I772M: 10 female and 7 male mice) as described previously (42). In total, 120 μm–thick slices were cut in ice-cold bicarbonate-buffered saline (BBS) (100 mmol/L NaCl, 2 mmol/L KCl, 26 mmol/L NaHCO_3_, 0.1 mmol/L CaCl_2_, 5 mmol/L MgCl_2_, 10 mmol/L glucose, 10 mmol/L HEPES) and continuously gassed with carbogen (95% O_2_ + 5% CO_2_). Staining was performed for 1 hours with Fura-2 AM or Calbryte 520 AM in BBS. See Supplemental Methods for more information.

### Behavioral phenotyping.

All animals were habituated to handling by the experimenters for at least 2 weeks prior to behavior testing. For isradipine treatment, mice were randomly assigned to 2 different groups, and experimenters were blinded to the 2 groups but not genotype. In isradipine treatment, mice were given sweetened yogurt supplemented with 12.5 mg/kg isradipine once daily. Vascal uno (5 mg) capsules (CHEPLAPHARM Arzneimittel GmbH) containing a slow-release formulation of isradipine were opened, the powder was mixed with sweetened yogurt and weighed for dosing. In the control group, mice were given sweetened yogurt once daily.

The general health status of the mice was assessed by a SHIRPA test modified from (64). Phenotyping included the Rotarod test, open-field test (64), novel object recognition, 3-chamber test, nest construction test (64), buried food test (64), social proximity test, and HCS over 24 hours (64, 65). We also performed mating behavior monitoring (66). The behavior was recorded with a video camera and assessed as described (67, 68). See Supplemental Methods for more detailed information on the performed tests and their analysis.

### qPCR.

Total RNA from kidneys and adrenal glands was isolated using the RNeasy Mini Kit (Qiagen) or NucleoSpin RNA Plus XS kit (Macherey-Nagel). Concentration and purity were determined using a Nanodrop 2000 (Thermo Fisher Scientific). Reverse transcription of RNA was performed using Quantitect RT Kit (Qiagen), followed by TaqMan gene expression assays (Applied Biosystems) for *Gapdh* (Mm99999915_g1, housekeeping gene), *Cyp11b2* (Mm00515624_m1), or *Ren1* (Mm02342887_mH) for adrenal and kidney samples. Gene expression was evaluated relative to *Gapdh* and mean ΔCT of WT controls, and it was expressed as 2^ΔΔCt^ (fold change). 

### Aldosterone, renin, and insulin ELISAs.

Plasma aldosterone, renin, and insulin concentrations were measured by ELISA (aldosterone [RE52301, IBL International]; renin [SEA889Mu, Cloud-Clone Corp.]; insulin [10-1247-01, Mercodia]) according to the manufacturers’ instructions.

### Histology and IHC.

H&E staining of 5 μm–thick slices of FFPE organs was performed as described (43). Nissl staining of sagittal FFPE brain sections was performed after the following steps: 7 minutes in xylene (3×), 3 minutes in 100% ethanol (3×), 3 minutes in 95% ethanol (1×), 3 minutes in 80% ethanol (1×), and 5 minutes in deionized H_2_O (1×).

Staining was performed by immersion of slides in 0.5% cresyl violet stain solution (ab246817, Abcam) for 2 minutes. Afterward, slides were immersed in 95% ethanol for 2 minutes and 2× in 100% ethanol for 3 minutes each, followed by 3 minutes in xylene (2×). Samples were mounted in xylene-based solution (Permount) and dried overnight at room temperature.

### ISH.

Detection of mRNA in organ sections was performed using the RNAscope 2.5 HD Assay – Brown (Advanced Cell Diagnostics) according to the manufacturer’s instructions. Probes used were: Mm-Ppib (positive control; 313911), DapB (negative control; 310043), Mm-Cyp11b2 (catalog 505851), and Mm-Th (catalog 317621).

### Microscopy and image analysis.

Images were taken on a Keyence BZ-9000 microscope. Images were processed, stitched, and analyzed using Fiji (69). See Supplemental Methods for more information.

### Electrolytes and isradipine levels.

Serum and urine electrolytes and glucose levels were determined as described (42). Isradipine levels were determined by liquid chromatography–tandem mass spectrometry (LC-MS/MS). See Supplemental Methods for more information.

### Statistics.

Statistical analysis was performed using Python (3.9) and the Scipy library (70) (version 1.8.0) except for mixed model analysis, which was performed in R (3.6.0) using either the lme4 (71) (version 1.1-21) package for Fura-2 AM calcium imaging or glmmTMB (version 1.1.3) for HCS and Calbryte 520 AM calcium imaging data. 

Two-tailed *t* test was used if normality of the sample distributions was likely based on a Shapiro-Wilk test calculated by the Scipy.stats.shapiro function. We chose to compare groups using a 2-tailed *t* test calculated by the Scipy.stats.ttest_ind function. Results are reported with the *t* statistic (*t*) and the associated *P* value.

Mann-Whitney *U* test was used if normality was not given based on a Shapiro-Wilk test calculated by the Scipy.stats.shapiro function. We chose to compare groups using the Mann-Whitney *U* test as calculated by the Scipy.stats.mannwhitneyu function. Results are reported with the *U* statistic (*U*) and the associated *P* value.

Fisher’s exact test was calculated using the Scipy.stats.fisher_exact function. Results are reported with the calculated *P* value.

Log rank test was calculated using the log_rank function of the lifelines library (version 0.27.7). Within this test, significance was either determined by calculation of a χ^2^ or *z* statistic (*z*). For the former, results are reported with the χ^2^ statistic, the degrees of freedom (df), and the associated *P* value. Results from the latter are reported with the *z* statistic and the associated *P* value.

Likelihood ratio test of linear mixed models was used to investigate differences between genotypes in a nested structure (typically in cases where multiple recordings for each animal were taken). We compared linear mixed models including and excluding the genotype in R using the anova or summary functions. Significance is calculated based on the resulting χ^2^ statistic for 1 df. Results are reported with the χ^2^ statistic and the associated *P* value.

For all tests, *P* values below 0.05 were considered significant. Where shown, 95% CI values of the mean were calculated by resampling using the Bootstrap procedure for 10,000 times. All box plots follow Tukey style with the box showing 25th (upper box limit), median (central horizontal line), and 75th percentiles (lower box limit). Whiskers reach to the maximum and minimum values within a range of 1.5***×*** IQR (distance between 25th and 75th percentiles), and values outside of this range are shown as diamonds. Please see Supplemental Methods for more information.

### Study approval.

Animal studies were approved by the Landesamt für Gesundheit und Soziales (LaGeSo) Berlin under G0210/17, G0095/20, and T0425/17.

### Data availability.

Values of individual data points underlying figures and mean values presented in this paper can be found in the accompanying Supporting Data Values file. Further raw data and the Python scripts used to generate the figures shown in this publication can be found at https://doi.org/10.5281/zenodo.7895995 The full raw video files from behavioral testing as well as from calcium imaging can only be provided upon reasonable request due to their file sizes.

## Author contributions

UIS conceived the project; GS, HAD, MV, LH, JS, and UIS performed phenotyping and treatment; ML and MR trained and supervised phenotyping; GS, HAD, and MV performed calcium imaging. GS, HAD, MV, and LH performed analyses. GS, LH, and NH performed histology, IHC, and ISH; HO measured isradipine in mouse plasma; JZ and RPL maintained colonies; ; IU planned and performed IVF; NH, JS, and ES performed genotyping; TN generated the mouse model; GS and UIS wrote the initial draft of the manuscript, with all authors contributing to the final version. The order of co–first authors was determined based on their time spent on this project.

## Supplementary Material

Supplemental data

Supplemental video 1

Supplemental video 2

Supplemental video 3

Supplemental video 4

Supplemental video 5

Supporting data values

## Figures and Tables

**Table 4:**
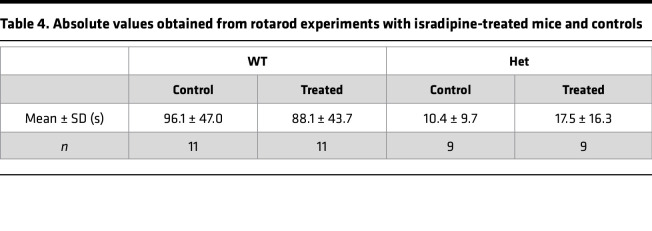
Absolute values obtained from rotarod experiments with isradipine-treated mice and controls

**Table 3:**
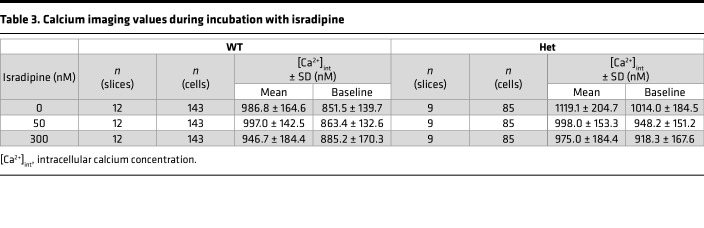
Calcium imaging values during incubation with isradipine

**Table 1:**
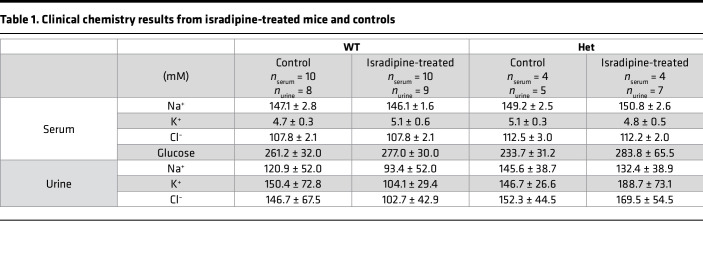
Clinical chemistry results from isradipine-treated mice and controls

**Table 2:**
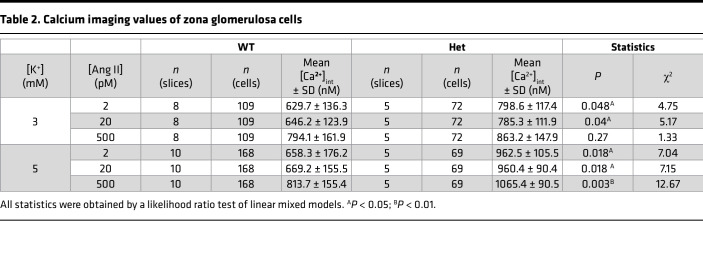
Calcium imaging values of zona glomerulosa cells

## Version 1 09/12/2023

In-Press Preview

## Version 2 10/23/2023

Electronic publication
