# Supplementary material for: Isradipine therapy in Cacna1dIle772Met/+ mice ameliorates primary aldosteronism and neurologic abnormalities
Source: JCI Insight. 2023 Oct 23;8(20):e162468. doi: 10.1172/jci.insight.162468 (PMC10619505; doi:10.1172/jci.insight.162468)
Supplement: Supplemental data [file jciinsight-8-162468-s057.pdf]

**Supplemental Information to**

# *Isradipine therapy in Cacna1d$^{Ile772Met/+}$ mice with PASNA syndrome (primary aldosteronism, seizures and neurologic abnormalities)*

Gabriel Stölting[1#], Hoang An Dinh[1#], Marina Volkert[1#], Nicole Hellmig[1], Julia Schewe[1], Luise Hennicke[1], Eric Seidel[1], Herbert Oberacher[2], Junhui Zhang[3], Richard P. Lifton[3,4], Iris Urban[5], Melissa Long[6], Marion Rivalan[6], Timothy Nottoli[7], and Ute I. Scholl[1*]

[1]Center of Functional Genomics, Berlin Institute of Health at Charité – Universitätsmedizin Berlin, Berlin, Germany.

[2]Institute of Legal Medicine and Core Facility Metabolomics, Medical University of Innsbruck, Innsbruck, Austria

[3]Department of Genetics and Howard Hughes Medical Institute, Yale University School of Medicine, New Haven, USA.

[4]Laboratory of Human Genetics and Genomics, The Rockefeller University, New York, USA.

[5]Transgenic Technologies, Charité – Universitätsmedizin Berlin, Berlin, Germany.

[6]Animal Behavior Phenotyping Facility (ABPF), Charité – Universitätsmedizin Berlin, Berlin, Germany

[7]Section of Comparative Medicine, Yale Genome Editing Center, Yale University School of Medicine, New Haven, USA.

(# shared first authors)

**Supplementary Methods**

**Generation and propagation of the *Cacna1d*$^{I772M/+}$ Mouse Model.** CRISPR/Cas9-mediated mutagenesis in the *Cacna1d* gene (Ile772Met) was performed at the Yale Genome Editing Center as described previously (42) using fertilized eggs from C57BL/6J mice. The protospacer sequence 5'-AGCCAAATTGTCCACAGCGATGG-3' (reverse strand) and a repair template (5'-ACACGTTGTACCCTTAAAGATTTCCAAATTAGATATCCTGCTGAATGTCTTCT TGGCCATGGCTGTGGACAATTTGGCTGATGCTGAAAGTCTGAACACTGCTC AGAAAGAGGAAGCTGAA-3'; mutant codon underlined) were used. For genotyping, DNA was extracted from ear biopsies, a 283 bp sequence was amplified using PCR (forward primer: 5'-TCAGGACATATGGCTGGACA-3'; reverse primer: 5'-TTACCCACTCCCAGCCTATG-3') and Sanger sequenced. Amplification and Sanger sequencing of *Cacna1d* exon 16 in adrenal cDNA was performed using the following primers: forward 5'-TTTCCGCAAGCACTCCTGACGG-3', reverse 5'-ACCTACTGGCACATCACAGGGC-3'. Sanger sequencing of the top 7 predicted off-target sites did not reveal any mutations. Experimental mice were housed at the FEM and maintained under specific-pathogen free conditions in a 12-h light/dark cycle with *ad libitum* access to food and water. All animal experiments were approved by the local authorities (Landesamt für Gesundheit und Soziales Berlin) and performed under consideration of all relevant ethical regulations.

**High-salt diet.** We fed mice with food pellets containing 1.71% $Na^+$ (4% NaCl; EF15431-347, Ssniff Spezialdiäten) *ad libitum* for two weeks. Drinking water was supplemented with 1% NaCl (w/v) with *ad libitum* access. Mice were habituated to handling during these two weeks. Afterwards, samples were collected as described below.

**IVF.** IVF was performed at the Yale Genome Editing Center and the Transgenic Technologies Core Facility within the Forschungseinrichtungen für Experimentelle Medizin (FEM, Charité - Universitätsmedizin Berlin) using C57BL/6J females as egg donors and sperm from male *Cacna1d*$^{I772M/+}$ mice. IVF was performed with either prepubertal donors (20-28 d) or fully matured donors (56-120 d). Females were superovulated by intraperitoneal injection of PMSG (Intergonan®, Intervet, Unterschleißheim, Germany, 5 IU, 8 p.m.,) and hCG (Ovogest®, Intervet, 2.5 IU, 6 p.m.) 46 h apart. 12-14 h post hCG, the oocytes were collected after sacrificing mice by cervical dislocation. The oocytes were co-incubated with capacitated fresh epididymal spermatozoa for 4 to 5 h in an incubator (CB 60, Binder, Austria) at 37°C in 5% $CO_2$. After removal of the sperm by washing with K-RVFE (K-RVFE, Cook Medical Europe, Baesweiler, Germany), the oocytes were incubated overnight in an incubator at 37°C in 5% $CO_2$. After another 20 h, the morphologically intact 2-cell embryos with 2 blastomeres of approximately the same size with homogeneous cytoplasm and intact zona were selected under a stereo microscope (Leica MZ12.5, Leica, Germany), followed by embryo transfer. Sperm harvest and embryo transfer were performed as published (62).

**Sample collection.** To harvest organs for RNA extraction and histology, mice were either anesthetized using ketamine (100 mg/kg) + xylazine (10 mg/kg) i.p. or isoflurane (via vaporizer set to 5% at 1 l/min oxygen flow). Euthanasia was performed using cervical dislocation or terminal blood collection by cardiac puncture. To determine renin concentrations, the first 100 µl of blood were collected into tubes containing 10 µL EDTA (0.1 M). The remaining blood was collected into Vacutainer EDTA tubes (Becton Dickinson). Blood samples were centrifuged (10 min, 2000g, 4 °C), and the supernatant (plasma) was stored at -20 °C until further analysis. Urine samples were taken postmortem from the bladder using a syringe and stored at -20 °C until further analysis.

Organs for RNA extraction were stored in tubes containing RNAlater (Sigma Aldrich; 4 °C overnight, then -20 °C). For histology, organs were rinsed in PBS and fixed in 10% formalin solution (neutral buffered; Sigma Aldrich) for 18–24 h. All samples were dehydrated in ethanol (70 %, 80 %, 96 %, and 100 %) and xylene, followed by embedding into paraffin (Merck Millipore) and stored for further investigations.

**Adrenal calcium imaging.** For acute slice preparations, adrenal glands were extracted from 10- to 18-wk-old mice (WT: 7 female and 9 males; I772M: 10 female and 7 male mice) as described previously (42). Mice were anesthetized using isoflurane (400 µL as open drop in a 2-l beaker) and euthanized by cervical dislocation. Glands were transferred into ice-cold bicarbonate-buffered saline

(BBS: 100 mmol/L NaCl, 2 mmol/L KCl, 26 mmol/L $NaHCO_3$, 0.1 mmol/L $CaCl_2$, 5 mmol/L $MgCl_2$, 10 mmol/L glucose, 10 mmol/L HEPES) and continuously gassed with carbogen (95% $O_2$ + 5% $CO_2$) during all following steps. Surrounding fat was removed, glands embedded into 3% low-melting temperature agarose in BBS and glued to the mounting stage of a vibratome (7,000 smz-2; Campden Instruments). Slices were cut at 120 µm and transferred to BBS at 35 °C for 20 min. Slices were stored for up to 6 h in BBS with 2 mmol/L $CaCl_2$ (storage BBS) at room temperature (~22 °C). Staining was performed for 1 h in a cell culture insert in a 24-well plate at room temperature. One well was filled with 750 µL of BBS and the insert with 250 µL containing initially either 64 µmol/L Fura-2 AM + 0.16 % Pluronic F-127 or 37 µmol/L Calbryte 520 AM + 0.001 % Pluronic dissolved in storage BBS. With Fura-2 AM, this was followed by 15 min of de-esterification in storage BBS without Fura-2. Slices were placed in a recording chamber continuously perfused with solution from a reservoir supplied with carbogen gas and heated in line via a heating coil, resulting in a temperature of the perfusion solution of 33 ± 1 °C. Two solutions were prepared and mixed to yield the potassium concentration of interest, BBS 2K+ (100 mmol/L NaCl, 2 mmol/L KCl, 26 mmol/L $NaHCO_3$, 2 mmol/L $CaCl_2$, 1 mmol/L $MgCl_2$, 10 mmol/L glucose, 10 mmol/L HEPES and 5 mmol/L NaGluconate) and BBS 7K+ (same as BBS 2K+ with NaGluconate replaced with 5 mmol/L KGluconate). Ang II was added at the desired concentrations. Fura-2 was alternately excited at 340 and 385 nm using a FuraLED light source (Cairn Research). Calbryte was excited at 470 nm. Images were taken every 100 ms with 10-ms exposure using an OptiMOS camera (QImaging).

**Behavioral Phenotyping.** All animals were habituated to handling by the experimenters for at least two weeks prior to behavior testing. Phenotyping was performed in three separate cohorts, the first two under native conditions (WT: 11 female, 8 male; Het: 9 female, 11 male) and a third cohort for the treatment with isradipine (WT: 11 female, 12 male; Het: 9 female, 9 male). To track movement in cages, animals of the first two cohorts had a wireless RFID transponder subcutaneously implanted using a delivery needle (Euro I.D.) under 1-3% isoflurane anesthesia. One *Cacna1d*$^{Ile772Met/+}$ mouse died shortly after implantation. The general health status of the mice was assessed by a SHIRPA test modified from (63). In brief, the test comprises a set of 45 discrete measures of the animal's weight, appearance and activity, as well as morphological, affective, sensory, and motor function.

The first two cohorts of animals were evaluated in a set of tests over the age of 13-18 weeks in the following order: (1) untrained rotarod, (2) open field test, (3) novel object recognition test, (4) three-chamber test, (5) nest construction test, (6) buried food test, (7) social proximity test and (8) screening of natural behaviors in home cage. All tests were performed during the light phase (~2 hours after the dark cycle had ended) except for the nest construction test and the home cage screening test. Before starting behavioral tests, mice were allowed to habituate for at least 30 min in an adjacent room.

For the third cohort, 13-14 week old mice were randomly assigned to two different groups: (1) Isradipine treatment: Mice were given sweetened yogurt (330 mg, 9%

glucose) supplemented with 12.5 mg/kg isradipine once daily. Vascal uno 5 mg capsules (CHEPLAPHARM Arzneimittel GmbH) containing a slow-release formulation of isradipine were opened, the powder was mixed with sweetened yoghurt and weighed for dosing. (2) Control mice were given sweetened yogurt (330 mg, 9% glucose) once daily. The open field, rotarod and nest construction test were conducted either ~18 h (open field), ~4 h (rotarod), or immediately (nest construction) after yogurt/isradipine feeding. Treatment started one week before behavioral testing. Blood and organ samples were collected approx. 20 h following the last yogurt/isradipine feeding. **Randomization and Blinding.** Randomization of mice to the isradipine-treatment or control groups was performed via a custom python script. Experimenters were blinded to the treatment with preparation and feeding of mice with yoghurt (± isradipine) performed by separate personnel. Blinding to the genotype was not possible due to the phenotypic differences. Unblinding occurred after behavioral testing had been completed. **Untrained rotarod.** Motor skills were quantified using the Rotarod test (RotaRod Advanced, TSE Systems GmbH, Germany). Mice were placed on a rotating rod (set to 4 rpm). Once mice were able to successfully stay on the rod, the timer was started, and the rotational speed gradually increased to a maximum of 40 rpm after 5 minutes. The latency until fall was measured. The experiment was repeated 3 times with 15 minutes breaks in between trials. **Open field test.** The test was performed as described (63). In brief, the animal’s activity was assessed in a square arena (50 cm x 50 cm white box, 50 cm height). Individual mice were placed in the center of the open field and were allowed to explore it for up to 10 min. Mice were recorded

using a video camera placed above the arena. For mice in the isradipine-cohort, the duration was shortened to 6 minutes, by which time the difference was already clearly visible in the other cohorts. **Novel object recognition test.** The general setup was as for the open field test. During the first trial, mice were allowed to explore the empty arena for 10 minutes. For the second trial on the following day, two identical objects (small boxes) were placed inside of the box. The duration the mouse spent exploring either object over 10 minutes was recorded using a video camera and extracted using a video tracking software (Viewer, Biobserve). In the third trial (1 hour after 2$^{nd}$ trial), one of the two objects was replaced with a novel object (a spiky ball), and the time exploring each object over 5 minutes was taken. The discrimination ratio was calculated as (Duration $_{\text{at novel object}}$ – Duration $_{\text{at known object}}$) / (Duration $_{\text{at novel object}}$ + Duration $_{\text{at known object}}$). **Three-chamber test.** Mice were placed in an arena (60 x 40 x 22 cm, Stoelting apparatus) consisting of three adjacent chambers connected by openings in the separating transparent walls. During the first trial, mice were allowed to freely explore this arena for 10 minutes for habituation. For the second trial, a stranger mouse was placed in a small cage inside one of the outer chambers. A second empty cage was placed in the opposite outer chamber. The mouse was again placed in the setup for 10 minutes and left to freely explore and interact with the stranger mouse through the cage. After an intertrial interval of 5 minutes, for the final trial, another stranger mouse was placed in the previously empty cage in the opposite chamber of the now known mouse. The tested mouse was again placed in the arena for 10 minutes and left to explore. The setup was continuously observed using a video camera, and times spent in

each chamber and near the cages containing stranger mice were measured using Viewer software. Sniffing interactions were counted manually by the experimenters. **Nest construction test.** The test was performed as described (63). In brief, mice were placed in separate home-cages that included regular bedding, food and water *ad libitum* as well as a defined, previously weighed sheet of cellulose nesting material. After approximately 18 h, the structure of the built nest was assessed according to a scale from 0 to 5 and by weighing the remaining, unused nesting material. **Buried food test.** The test was performed as described (63). In brief, 16 h before testing (from 2 h before dark phase until 2 h after the dark phase), food was removed from the home-cages with water remaining *ad libitum*. The mouse was placed in the testing cage supplemented with a sweet pellet hidden 1 cm deep and 5 cm away from the cage rear in the 4 cm thick layer of fresh bedding. The latency to find the sweet pellet was recorded manually by experimenters. **Social proximity test.** The tested mouse and a stranger mouse were placed together into a small box (7 x 14 cm, transparent walls) and observed over a period of 10 minutes. Behavior was recorded by video camera. Two investigators separately assessed behavior by 7 criteria: nose tip-to-nose tip contacts, nose-to-head contacts, nose-to-anogenital contacts, crawl over events, crawl under events, uprighting or jumps. The averaged numbers between the two investigators were used for further analysis. **Home cage screen (HCS).** The HCS was performed as described (63, 64). In brief, mice were separated and single housed in a cage for 24 h. The behavior of the freely moving mouse was recorded with a video camera and automatically assessed with the HCS software

(CleverSys). **Mating behavior monitoring.** The tested male mouse was placed in a cage for 48 h together with a stranger female mouse in proestrus or estrus. Estrus was induced by adding soiled bedding of males to the cage of the female mice prior to the experiment. The stage of estrous cycle was determined by visual observation as described (65). The behavior was recorded with a video camera and assessed by 3 criteria: mounting, intromission with subsequent genital cleaning or ejaculation/successful copulation as described (66, 67).

**Microscopy and image analysis.** Images were taken on a Keyence BZ-9000 microscope using 10x/0.45 or 20x/0.75 objectives in color mode. Images were processed and stitched using Fiji (68). We wrote a custom macro that subtracted the background of each individual tile using the built-in rolling ball background subtraction (size=50), followed by stitching using the built-in Grid/Collection stitching plugin based on the known position of each tile (tile overlap: 20%, fusion method: Linear Blending, regression threshold: 0.3, max/avg displacement threshold: 2.5, absolute displacement threshold: 3.5).

For the analysis of ZG thickness, images were separated into different color channels using the color deconvolution feature (H&E DAB setting). Regions of adipose tissue were deleted, and thresholding (Otsu, automatic detection) was used on the DAB color channel to isolate regions with DAB signal. The local thickness plugin was used to determine the thickness of these regions. Using the wand selection tool, the outer regions (corresponding to the ZG) were selected, and thickness values were extracted and averaged for further statistical analysis.

For the analysis of nucleus size and density, the striatum from sagittal Nissl-stained brain sections was selected in Fiji, and thresholding (Otsu, automatic detection) was applied to select nuclei. The analyze particle function (size: 4-Infinity, circularity: 0.25 to infinity) was then used to quantify areas and number of particles. Brain ISH images were analyzed by first separating the DAB color channel as described above and then undergoing thresholding and particle analysis as for the Nissl-stained images.

**Electrolytes and isradipine levels.** Serum and urine electrolytes and glucose were determined as described (42). Isradipine levels were determined by liquid chromatography-tandem mass spectrometry (LC-MS/MS). An isotopically labeled analogue was used as internal standard (isradipine-D3). Water, methanol, acetic acid and acetonitrile (all HPLC grade) were purchased from Honeywell. Isradipine and Isradipine-D3 were obtained from Toronto Research Chemicals. The LC-MS/MS system consisted of a Waters ACQUITY UPLC H-Class PLUS Bio (Waters) and a QTrap 6500+ mass spectrometer (Sciex). Chromatographic separations were accomplished on a reversed-phase column (Kinetex 2.6 µm Biphenyl 100 Å, 100 × 2.1 mm, Phenomenex) using a 1-minute linear gradient of 10-98% methanol in aqueous 0.5% acetic acid solution (v/v). The gradient program was started 1 min after the injection. The flow rate was set to 200 µl/min. The column temperature was held at 50 °C. The injection volume was 10 µL. Mass spectrometry detection was performed with electrospray ionization in negative ion mode. Multiple reaction monitoring was carried out using the precursor-to-product

ion transitions *m/z* 370.1 to 250.1 and *m/z* 370.1 to 119.1 for isradipine and *m/z* 373.1 to 253.1 and *m/z* 373.1 to 119.1 for isradipine-D3. Sample preparation involved a protein precipitation step. Before use, plasma samples were allowed to equilibrate to room temperature. A 50 µL-aliquot of the sample was mixed with 5 µL of internal standard solution (30 ng/mL) and 95 µL of methanol. The mixture was vortexed and centrifuged at 4600 × g for 5 min. The obtained supernatant was submitted to LC-MS/MS analysis. Calibration was accomplished by donating 50 µL of isradipine-free mouse plasma with known concentrations of isradipine ranging from 0.020 to 100 ng/ml. The limit of quantification was 0.050 ng/ml.

**Statistics.** Statistical analysis was performed using Python (3.9) and the Scipy library (69) (version 1.8.0) except for mixed model analysis which was performed in R (3.6.0) using either the lme4 (70) (version 1.1-21) package for Fura-2 AM calcium imaging or glmmTMB (version 1.1.3) for HCS and Calbryte 520 AM calcium imaging data. Dichotomic data was compared using Fisher's exact test. For the comparisons of means or medians, the normality of the data distribution was assessed using the Shapiro-Wilk-Test (shaprio function in Scipy). If the normality assumption was not violated, samples were compared using a two-tailed Student's t-test (ttest_ind function in Scipy), otherwise a Mann-Whitney-U test was performed (mannwhitneyu function in Scipy) as indicated in the respective figure legend. For linear mixed model analysis of Fura-2 AM calcium imaging (lmer function of lme4), normality was assured visually using quartile-quartile plots and inspection of residuals after fitting. Home cage scan data and

calcium spiking activity data was not normally distributed, so a diverse set of distributions was tested using DHARMa (version 0.4.5), and the log-Tweedie distribution was chosen for further analysis using glmmTMB. P-values below 0.05 were considered significant. Where shown, 95% CI values of the mean were calculated by resampling using the Bootstrap procedure for 10,000 times. All box plots follow Tukey-style with the box showing 25$^{th}$ (upper box limit), median (central horizontal line) and 75$^{th}$ percentiles (lower box limit). Whiskers reach to the maximum and minimum values within a range of 1.5×IQR (inter-quartile range: distance between 25$^{th}$ and 75$^{th}$ percentiles) and values outside of this range are shown as diamonds.

## Supplementary Figures

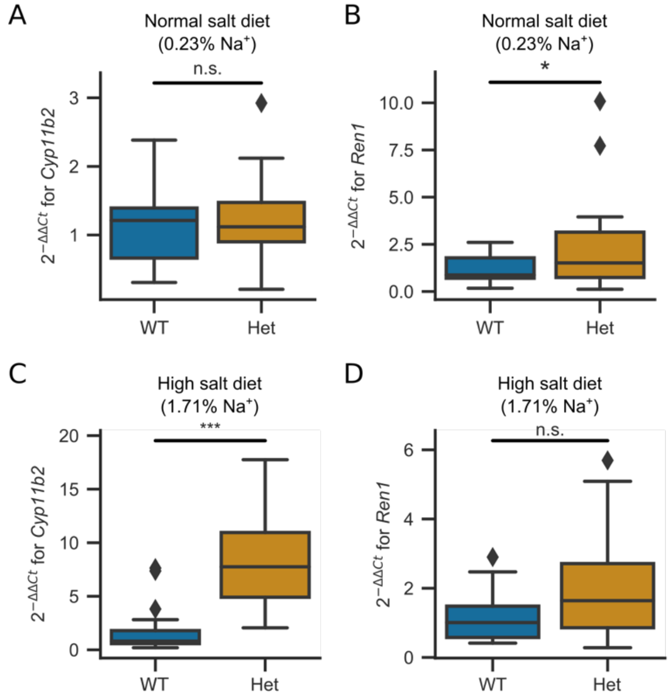


**Supplemental Figure 1: *Cyp11b2* and expression in adrenal glands and *Ren1* expression in kidneys of mice under normal-salt and high-salt diets.**

**(A)** *Cyp11b2* is not significantly elevated in *Cacna1d*$^{Ile772Met/+}$ mice (Het) compared with WT (Mann-Whitney-U; $n_{WT}$ = 24, $n_{Het}$ = 20, p = 0.60, U = 263). **(B)** *Ren1* expression is increased in knock-in mice (Mann-Whitney-U; $n_{WT}$ = 25, $n_{Het}$ = 20, p = 0.02, U = 354). **(C)** *Cyp11b2* is higher in *Cacna1d*$^{Ile772Met/+}$ mice (Het) compared with WT under a high-salt diet with 4% NaCl (1.71% $Na^+$) for 2 weeks (Mann-Whitney-U; $n_{WT}$ = 27, $n_{Het}$ = 26, p = $1.8\times10^{-8}$, U = 668). **(D)** Median *Ren1* expression under a high salt diet is not significantly elevated (Mann-Whitney-U; $n_{WT}$ = 20, $n_{Het}$ = 24, p = 0.052, U = 323). All panels show box plots (Tukey, see Methods).

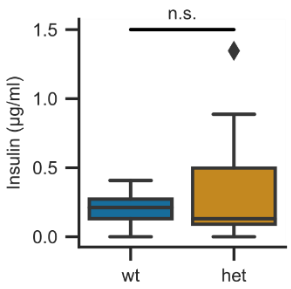


**Supplemental Figure 2: Insulin levels are similar in 14-week-old WT and *Cacna1d*$^{Ile772Met/+}$ mice.**

Insulin concentrations in plasma are not significantly different between genotypes (Mann-Whitney-U; $n_{WT}$ = 18, $n_{Het}$ = 10, p = 0.98, U = 89). Shown are box plots (Tukey, see Methods).

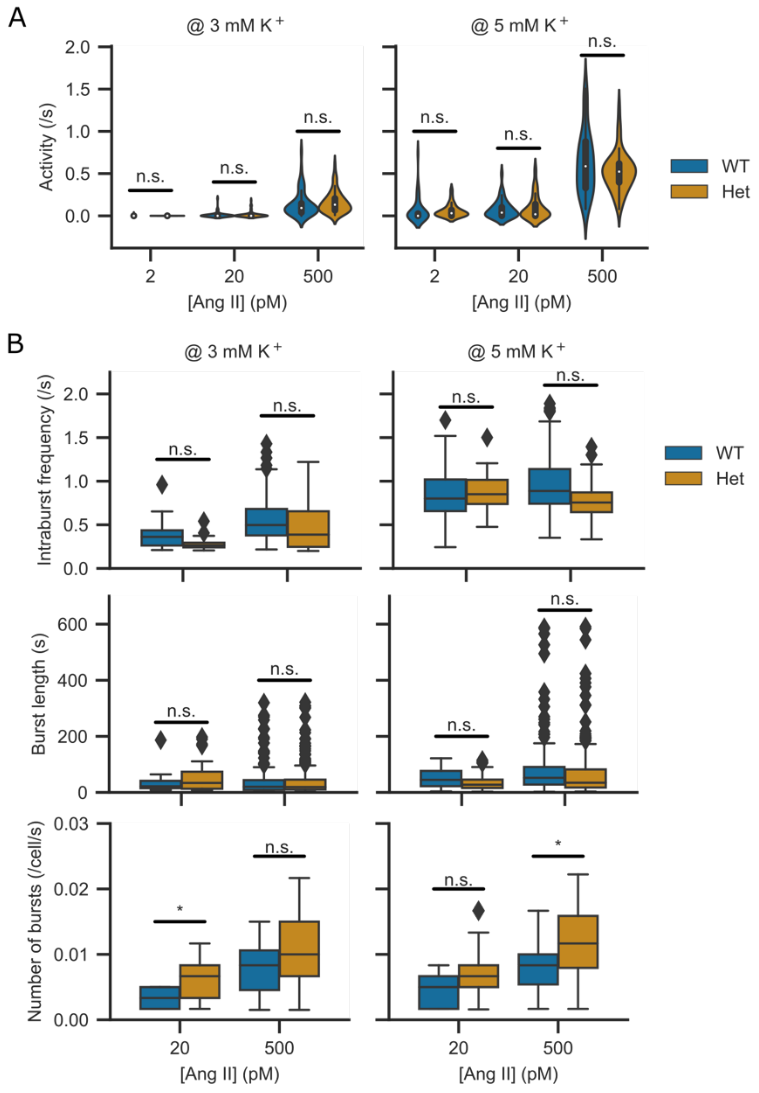


**Supplemental Figure 3. *Cacna1d*$^{Ile772Met/+}$ mice show calcium spiking frequencies similar to WT mice. (A)** Both, WT and *Cacna1d*$^{Ile772Met/+}$ mice, show increases in spiking frequencies in acute adrenal slice preparations upon increasing potassium and raising angiotensin to supra-physiologic levels (500 pM). However, no difference in activity exists between WT and *Cacna1d*$^{Ile772Met/+}$ mice (Generalized linear mixed model; @ 3 mM $K^+$: $n_{WT}$ = 56 cells/4 animals, $n_{Het}$ = 78 cells/4 animals; p(2/20/500 pM Ang II) = 0.11/0.20/0.67, $\chi^2$ = 2.58/1.61/0.18;

@ 5 mM $K^+$: $n_{WT}$ = 40 cells/4 animals, $n_{Het}$ = 56 cells/4 animals; p(2/20/500 pM Ang II) = 0.25/0.74/0.69). For better temporal resolution, the intensiometric calcium-sensitive dye Calbryte 520 AM was used for this experiment. Violin plots are are generated via kernel density estimation using a gaussian kernel. Boxes embedded in each individual violin plot follow Tukey-style (see Methods). (B) Analysis of bursting parameters yields a heterogeneous picture. No differences were observed for the intra-burst frequency (Generalized linear mixed model; @ 3 mM $K^+$: $n_{WT}$ = 56 cells/4 animals, $n_{Het}$ = 78 cells/4 animals; p(20/500 pM Ang II) = 0.19/0.86, $\chi^2$ = 1.71/0.03; @ 5 mM $K^+$: $n_{WT}$ = 40 cells/4 animals, $n_{Het}$ = 56 cells/4 animals; p(20/500 pM Ang II) = 0.98/0.23, $\chi^2$ = $9\times10^{-4}$/1.42) and burst length (Generalized linear mixed model; @ 3 mM $K^+$: $n_{WT}$ = 56 cells/4 animals, $n_{Het}$ = 78 cells/4 animals; p(20/500 pM Ang II) = 0.43/0.91, $\chi^2$ = 0.62/0.014; @ 5 mM $K^+$: $n_{WT}$ = 40 cells/4 animals, $n_{Het}$ = 56 cells/4 animals; p(20/500 pM Ang II) = 0.07/0.11, $\chi^2$ = 3.27/2.54). However, there was a general trend towards more bursts per second and cell which was significant for some conditions (Generalized linear mixed model; @ 3 mM $K^+$: $n_{WT}$ = 56 cells/4 animals, $n_{Het}$ = 78 cells/4 animals; p(20/500 pM Ang II) = 0.03/0.52, $\chi^2$ = 4.64/0.42; @ 5 mM $K^+$: $n_{WT}$ = 40 cells/4 animals, $n_{Het}$ = 56 cells/4 animals; p(20/500 pM Ang II) = 0.17/0.04, $\chi^2$ = 1.91/4.13). As the number of spikes is low at 2 pM Ang II, only the stimulation with 20 and 500 pM Ang II was analyzed. Box plots are shown as Tukey-style (see Methods).

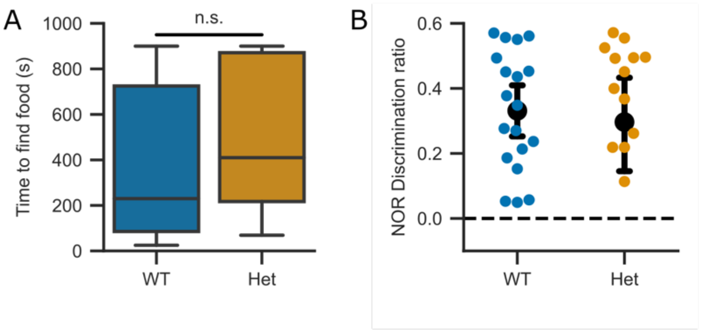


**Supplemental Figure 4: *Cacna1d*$^{\text{Ile772Met/+}}$ mice exhibit similar performance in buried food and novel object recognition tests.**

**(A)** Latency until finding a buried food pellet is similar between WT and knock-in mice (Mann-Whitney-U; $n_{WT}$ = 19, $n_{Het}$ = 18, p = 0.12, U = 120). Box plots (Tukey, see Methods). **(B)** Both genotypes show a positive novel object recognition discrimination ratio (mean: black circle, whiskers: 95% CI as computed by 10,000 bootstrap resamples; individual values are shown as colored circles). The ratio is also similar between WT and knock-in mice (two-tailed t-test; $n_{WT}$ = 19, $n_{Het}$ = 19, p = 0.69, t = 0.40) and both are above .

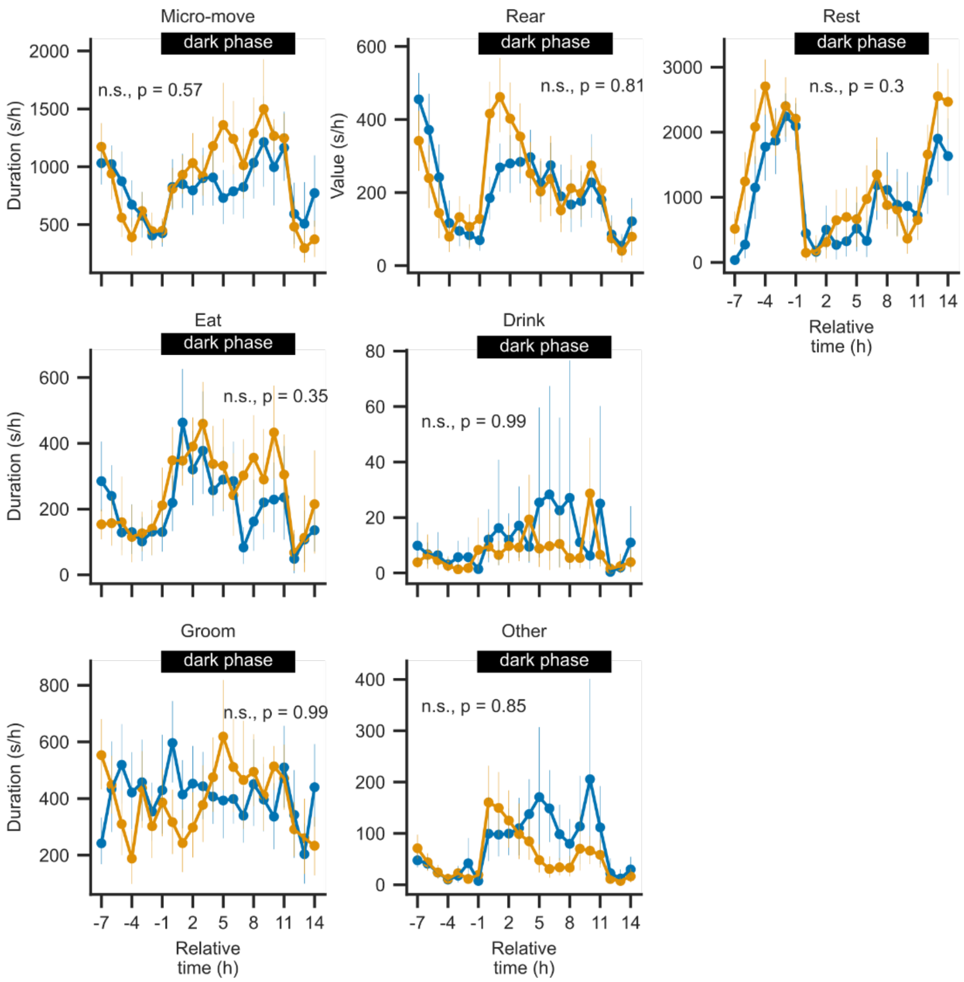


**Supplemental Figure 5: Overview of further parameters automatically analyzed in home cage scan.**

Home-cage behaviors that were grouped according to (64) and that are not shown in Fig. 7. Black bar indicates dark period. All comparisons are based on generalized linear mixed models. Sample sizes are always $n_{WT}$ = 19, $n_{Het}$ = 17, and p-values are adjusted for 10 comparisons using the FDR method. Micro-move: p = 0.57, t = -0.95; Rear: p = 0.81, t = -0.577; Rest: p = 0.3, t = -1.56; Eat: p = 0.35, t = -1.36; Drink: p = 0.99, t = 0.085; Groom: p = 0.99, t = -0.01; Other (this is labeled

“unknown behavior” in the original publication): $p = 0.85$, $t = 0.414$. Data are shown as mean ± 95% CI

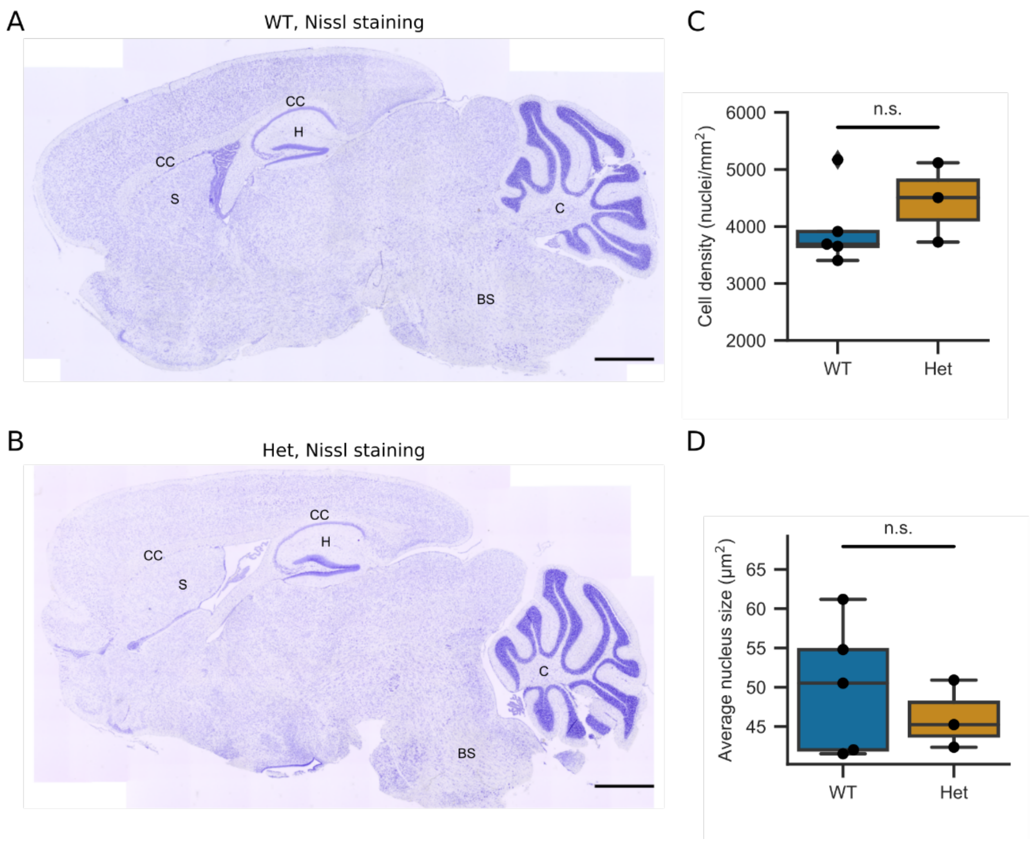


**Supplemental Figure 6: Brain morphology is similar between genotypes.**

**(A-B)** Representative images of Nissl-stained sagittal brain sections from 14-week old WT and *Cacna1d*$^{Ile772Met/+}$ mice. Brains were formalin-fixed, embedded in paraffin and cut in 5 µm slices. CC – corpus callosum, S – dorsal striatum, H – hippocampus, C – cerebellum, BS – brain stem. Scale bar, 1000 µm. **(C)** Analysis of the cell density in the striatum as assessed by automated detection of nuclei (Mann-Whitney-U; $n_{WT}$ = 5, $n_{Het}$ = 3, p = 0.39, U = 4). **(D)** Average nucleus size as a surrogate for the cell size is similar between genotypes (Mann-Whitney-U; $n_{WT}$ = 5, $n_{Het}$ = 3, p = 1.0, U = 8). (**C**) and (**D**) show box plots (Tukey, see Methods).

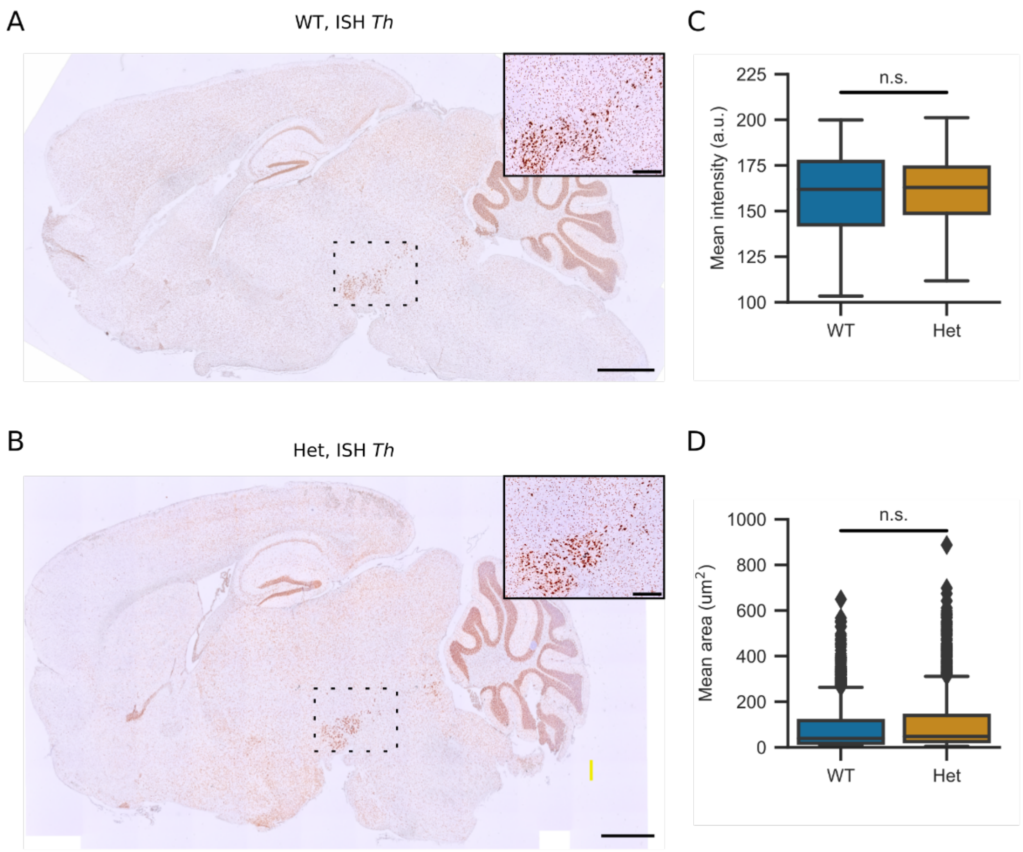


**Supplemental Figure 7: In situ hybridization for tyrosine hydroxylase (*Th*) in mouse brain.**

**(A-B)** Representative images of brain FFPE sections from WT or *Cacna1d*$^{Ile772Met/+}$ mice (scale bar 1000 µm). ISH was performed using the RNAScope kit and a probe against tyrosine hydroxylase (*Th*). Midbrain neurons highly expressing *Th* are shown enlarged (inset, scale bar 250 µm). **(C)** Comparison of the mean *Th* signal per identified signal cluster reveals similar levels. (likelihood ratio test of generalized linear mixed models; $n_{WT}$ = 2384 clusters/4 mice, $n_{Het}$ = 1746 clusters/3 mice, p = 0.58, $\chi^2$ = 0.3095) **(D)** The mean area per signal cluster is also similar between WT and *Cacna1d*$^{Ile772Met/+}$ mice, further supporting that expression of *Th* is generally similar between WT and Het (likelihood ratio test of generalized

linear mixed models; $p = 0.09$, $\chi^2 = 2.85$). (**C**) and (**D**) show box plots (Tukey, see Methods).

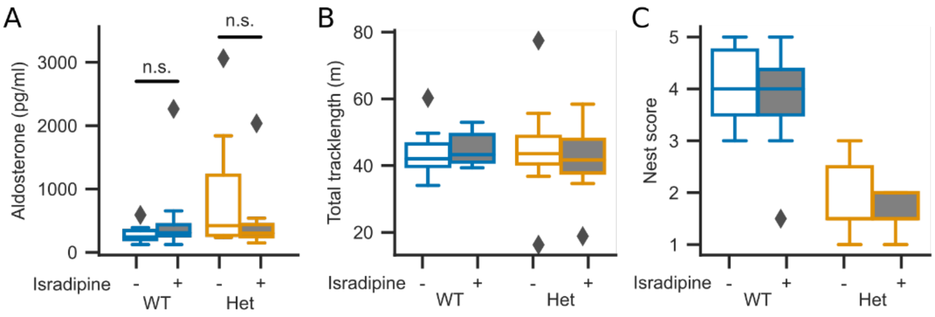


**Supplemental Figure 8: No effect of isradipine on locomotion at trough plasma levels and on nest building performance.**

**(A)** Serum aldosterone concentrations for treated (+) and untreated (-) WT or *Cacna1d*$^{Ile772Met/+}$ mice (Het). The absolute difference between treated and untreated controls is not significant (Mann-Whitney-U test; all given as untreated/treated: $n_{WT}$ = 11/11, $p_{WT}$ = 0.15, U=34; $n_{Het}$ = 9/9, $p_{Het}$ = 0.43, U=50). **(B)** Results from open field experiments with WT or Het mice either treated (+) or untreated (-) (18 h after last dose). Total tracklengths over 6 minutes were similar between genotypes and treatment. **(C)** *Cacna1d*$^{Ile772Met/+}$ mice show no improvement in nest construction score when treated with isradipine. The experiment was started following administration of isradipine and ran for approximately 18h. All panels show box plots (Tukey, see Methods).

**Supplementary Video legends**

**Supplemental video 1: Representative example #1 of an intromission event of a WT mouse during mating behavior assessment.**

**Supplemental video 2: Representative example #2 of an intromission event of a WT mouse during mating behavior assessment.**

**Supplemental video 3: Representative example of an intromission event of a *Cacna1d*$^{Ile772Met/+}$ mouse during mating behavior assessment.**

**Supplemental video 4: Representative example of a copulation event of a WT mouse during mating behavior assessment.**

**Supplemental video 5: *Cacna1d*$^{Ile772Met/+}$ mice develop clonic-tonic seizures after injection with ketamine/xylazine.** The mouse was administered 100 mg/kg ketamine and 10mg/kg xylazine i.p. approximately 5 minutes prior to this recording. Similar behavior was observed in 10 of 13 *Cacna1d*$^{Ile772Met/+}$ mice.

## Supplementary Tables

## Supplemental Table 1: Summary of *Cacna1d* mating behavior over 48 h.

| | | WT #1 | WT #2 | WT #3 | Het #1 | Het #2 | Het #3 |
|---|---|---|---|---|---|---|---|
| **0 - 24 h** | **Mounting** | 130 | 104 | 125 | 79 | 56 | 95 |
| | **Intromission** | 24 | 57 | 17 | 54 | 0 | 2 |
| | **Copulation** | 0 | 1 | 0 | 0 | 0 | 0 |
| **24 – 48 h** | **Mounting** | 36 | 0 | 5 | 4 | 20 | 14 |
| | **Intromission** | 15 | 0 | 0 | 28 | 0 | 5 |
| | **Copulation** | 0 | 0 | 0 | 0 | 0 | 0 |
| ***Sum*** | ***Mounting*** | *166* | *104* | *130* | *83* | *76* | *109* |
| | ***Intromission*** | *39* | *57* | *17* | *82* | *0* | *7* |
| | ***Copulation*** | *0* | *1* | *0* | *0* | *0* | *0* |

**Supplemental Table 2: Organ weights of 14-week old WT and *Cacna1d*$^{Ile772Met/+}$ mice**

| | | | Heart | Kidney | Brain | Pancreas |
|---|---|---|---|---|---|---|
| WT | f | **Mean ± s.d. (mg)** | 155 ± 48 | 263 ± 104 | 479 ± 48 | 301 ± 126 |
| | | **n** | 21 | 20 | 14 | 11 |
| | m | **Mean ± s.d. (mg)** | 195 ± 43 | 340 ± 110 | 464 ± 71 | 353 ± 108 |
| | | **n** | 20 | 20 | 17 | 11 |
| Het | f | **Mean ± s.d. (mg)** | 140 ± 30 | 209 ± 76 | 466 ± 31 | 216 ± 68 |
| | | **n** | 17 | 16 | 15 | 9 |
| | | **p** | 0.42 | 0.07 | 0.23 | 0.16 |
| | | **U** | 206.5 | 217 | 133 | 68.5 |
| | m | **Mean ± s.d. (mg)** | 149 ± 36 | 272 ± 86 | 473 ± 42 | 236 ± 42 |
| | | **n** | 15 | 15 | 11 | 9 |
| | | **p** | 8 x 10 4 (***) | 0.03 (*) | 0.64 | 0.02 (*) |
| | | **U** | 250.5 | 215.5 | 83 | 87.5 |

f, female; m, male. *, p<0.05; ***, p<0.001 (Mann-Whitney-U test).

**Supplemental Table 3: Full results of the SHIRPA test.** Scores are based on Schuster et al.(63) Statistical significance was either assessed using Fisher's exact test (for dichotomic scores, only p is stated), Student t-test (p and t are stated) or Mann-Whitney-U tests (p and U are stated). FDR adjusted p-values (43 subtests) are shown, and values below the alpha level of 0.05 are marked in bold.

| Test | Distribution of scores | p value | FDR adjusted p value | t | U |
|---|---|---|---|---|---|
| **Body position** | WT: score 1: 39, score 2: 3<br>Het: score 0: 1, score 1: 31, score 2: 5 | 0.59872 | >0.99999 | | 747 |
| **Limb morphology** | all 0 | | | | |
| **Tail morphology** | all 0 | | | | |
| **Head morphology** | all 0 | | | | |
| **Whisker morphology** | all 0 | | | | |
| **Skin color of plantar surface** | all 1 | | | | |
| **Hair length** | WT: score 0: 39, score 1: 3<br>Het: score 0: 35, score 1: 2 | 0.76202 | >0.99999 | | 790.5 |
| **Hair morphology** | all 0 | | | | |
| **Piloerection** | WT: score 2: 24, score 4: 17, score 6: 1<br>Het: score 0: 1, score 2: 29, score 4: 5, score 6: 1 | 0.01231 | 0.07735 | | 960 |
| **Ear morphology** | all 0 | | | | |
| **Eye characteristics** | WT: score 0: 40, score 1: 2<br>Het: score 0: 36, score 1: 1 | >0.99999 | >0.99999 | | |
| **Lacrimation** | all 0 | | | | |
| **Salivation** | all 0 | | | | |
| **Abnormal physical features** | all 0 | | | | |

| | | | | | |
|---|---|---|---|---|---|
| **Pinna reflex** | all 0 | | | | |
| **Corneal reflex** | all 0 | | | | |
| **Abnormal and stereotyped behavior** | WT: score 0: 42<br>Het: score 0: 31, score 1: 6 | 0.00619 | 0.0545 | -<br>2.81 | |
| **Convulsions** | all 0 | | | | |
| **Tremors** | WT: score 0: 40, score 1: 2<br>Het: score 0: 1, score 1: 36 | <0.00001 | **<0.00001** | | |
| **Twitches** | WT: score 0: 42<br>Het: score 0: 36, score 1: 1 | 0.46835 | >0.99999 | | |
| **Locomotor activity** | Mean ± s.d. WT: 19.4 ± 4.9<br>Mean ± s.d. Het: 17.8 ± 6.6 | 0.20706 | 0.70083 | 1.27 | |
| **Transfer arousal** | WT: score 0: 26, score 1: 16<br>Het: score 0: 23, score 1: 14 | 0.98601 | >0.99999 | | 779 |
| **Spatial locomotion** | WT: score 0: 36, score 1: 6<br>Het: score 0: 26, score 1: 11 | 0.09909 | 0.43598 | | 657 |
| **Gait** | WT: score 0: 42<br>Het: score 0: 34, score 1: 1, score 2: 2 | 0.07221 | 0.35302 | -<br>1.82 | |
| **Pelvic elevation** | WT: score 0: 33, score 1: 9<br>Het: score 0: 19, score 1: 18 | 0.01163 | 0.07735 | | 565.5 |
| **Tail elevation** | WT: score 0: 33, score 1: 9<br>Het: score 0: 27, score 1: 10, | 0.56824 | >0.99999 | | 733.5 |
| **Object approach** | WT: score 0: 42<br>Het: score 0: 36, score 1: 1 | 0.28959 | 0.84947 | -<br>1.07 | |
| **Vibrissa placing response when touched** | WT: score 0: 42<br>Het: score 0: 33, score 2: 4 | 0.02883 | 0.15856 | -<br>2.23 | |
| **Back touch escape** | WT: score 0: 8, score 1: 33, score 3: 1 | 0.67446 | >0.99999 | | 743.5 |

| | | | | | |
|---|---|---|---|---|---|
| | Het: score 0: 8, score 1: 25, score 3: 4 | | | | |
| **Acoustic startle response** | WT: score 1: 42<br>Het: score 0: 1, score 1: 33, score 2: 3 | 0.28959 | 0.84947 | -1.07 | |
| **Cage movement** | all 2 | | | | |
| **Limb grasping** | all 0 | | | | |
| **Visual placing** | all 0 | | | | |
| **Inclined plane** | WT: score 0: 42<br>Het: score 0: 36, score 1: 1 | 0.46835 | >0.99999 | | |
| **Grip strength** | WT: score 0: 38, score 1: 2, score 2: 2<br>Het: score 0: 23, score 1: 12, score 2: 2 | 0.00485 | 0.05339 | | 567 |
| **Air righting reflex** | all 0 | | | | |
| **Wire maneuver** | WT: score 0: 41, score 2: 1<br>Het: score 0: 19, score 2: 13, score 4: 2, score 6: 2, score 8: 1 | <0.00001 | **0.00003** | | 415 |
| **Positional passivity** | all 0 | | | | |
| **Provoked freezing** | WT: score 0: 41, score 1: 1<br>Het: score 0: 33, score 1: 4 | 0.12999 | 0.51997 | | 711.5 |
| **Vocalization** | WT: score 0: 40, score 1: 2<br>Het: score 0: 35, score 1: 2 | >0.99999 | >0.99999 | | |
| **Urination** | Mean ± s.d. WT: 0.4 ± 0.6<br>Mean ± s.d. Het: 0.2 ± 0.5 | 0.1473 | 0.5401 | | 887.5 |
| **Defecation** | Mean ± s.d.WT: 2.9 ± 2.2<br>Mean ± s.d. Het: 0.7 ± 1.5 | <0.00001 | **0.00001** | | 1280.5 |
| **Diarrhea** | all 0 | | | | |

| | | | | | |
|---|---|---|---|---|---|
| **Acute death** | all 0 | | | | |
